# ITS non-concerted evolution and rampant hybridization in the legume genus *Lespedeza* (Fabaceae)

**DOI:** 10.1038/srep40057

**Published:** 2017-01-04

**Authors:** Bo Xu, Xiao-Mao Zeng, Xin-Fen Gao, Dong-Pil Jin, Li-Bing Zhang

**Affiliations:** 1 CAS Key Laboratory of Mountain Ecological Restoration and Bioresource Utilization & Ecological Restoration and Biodiversity Conservation Key Laboratory of Sichuan Province, Chengdu Institute of Biology, Chinese Academy of Sciences, P.O. Box 416, Chengdu, Sichuan 610041, China; 2 Department of Biological Sciences, Inha University, Incheon 402-751, Republic of Korea; 3 Missouri Botanical Garden, P.O. Box 299, St. Louis, Missouri 63166, USA

## Abstract

The internal transcribed spacer (ITS) as one part of nuclear ribosomal DNA is one of the most extensively sequenced molecular markers in plant systematics. The ITS repeats generally exhibit high-level within-individual homogeneity, while relatively small-scale polymorphism of ITS copies within individuals has often been reported in literature. Here, we identified large-scale polymorphism of ITS copies within individuals in the legume genus *Lespedeza* (Fabaceae). Divergent paralogs of ITS sequences, including putative pseudogenes, recombinants, and multiple functional ITS copies were sometimes detected in the same individual. Thirty-seven ITS pseudogenes could be easily detected according to nucleotide changes in conserved 5.8S motives, the significantly lower GC contents in at least one of three regions, and the lost ability of 5.8S rDNA sequence to fold into a conserved secondary structure. The distribution patterns of the putative functional clones were highly different between the traditionally recognized two subgenera, suggesting different rates of concerted evolution in two subgenera which could be attributable to their different extents/frequencies of hybridization, confirmed by our analysis of the single-copy nuclear gene PGK. These findings have significant implications in using ITS marker for reconstructing phylogeny and studying hybridization.

Concerted evolution is a form of multigene family evolution in which all the tendency of the different genes in a gene family or cluster are assumed to evolve as a unit in concert^1^,^2^. All the repeats are maintained in the genome with very similar sequences but differ between related species. The best known example of concerted evolution is among multicopy nrDNA genes^3^. Nuclear ribosomal DNA cistron is arranged as tandemly repeated units consisting of 18S, ITS1, 5.8S, ITS2, 26S in all land plants except bryophytes, and occurs with hundreds to thousands copies per haploid genome^4^. The internal transcribed spacer (ITS1 and ITS2) as one part of nrDNA is one of the most extensively sequenced molecular markers in plant systematics because of the rapid concerted evolution within and among component subunits, fast evolution rate, and short length and availability of universal primers^5^,^6^,^7^,^8^. The ITS region generally undergoes rapid concerted evolution via unequal crossing over, high-frequency gene conversion, and large deletion^2^,^9^. Hence, intra-individual polymorphism has generally been considered to be an exception. Nevertheless, under certain circumstances, polymorphic ITS copies within individuals have been identified in many different plant groups but in a relatively small scale^10^,^11^,^12^,^13^,^14^,^15^,^16^,^17^,^18^,^19^,^20^,^21^,^22^,^23^,^24^ which suggest concerted evolution remains incomplete across the repeats. Various reasons such as hybridization, polyploidization, recombination among copies, and pseudogenization of cistrons are considered to be the causes of polymorphic ITS copies^5^,^17^,^24^,^25^. Among ITS polymorphism, non-functional pseudogenes are prominent and can be distinguished by the GC content, secondary structure stability, substitution rates, the presence of conserved motifs, and phylogenetic positions^6^,^19^,^21^,^22^.

Speciation via hybridization is thought to be an important evolutionary mechanism in plants^26^,^27^. Natural hybridizations are well documented and have been widely recognized in angiosperm evolution^26^,^27^,^28^,^29^, with an estimated approximately 25% of vascular plants forming hybrids with other species^30^ and about 11% of plant species probably being derived from past hybridization events^31^. The phenomenon of hybridization is particularly likely to occur in plant species complexes in which species are evolutionarily young and sometimes not well differentiated, and the barriers between them are relatively weak^32^,^33^,^34^,^35^,^36^,^37^. Hybridization leads the evolutionary processes at the species level to a network rather than a bifurcating tree, confounding efforts to reconstruct the phylogeny of the complexes. In such cases, the DNA sequence information of the uniparentally inherited plastid and that of the biparentally inherited nrDNA which often causes parents homogenization due to concerted evolution, mask evidence of reticulation in hybridizing species^38^,^39^,^40^,^41^. However, single or low-copy nuclear genes are able to overcome this disadvantage^32^,^42^. They are biparental and independently inherited and do not often experience concerted evolution, and thus can provide evolutionary signatures of unique gene copies from ancestral genomes which allows tracing hybrid reticulate history within lineages^42^,^43^,^44^. In addition, the direction of hybridization can be inferred when information of single or low-copy nuclear genes is used together with plastid markers.

The legume genus *Lespedeza* Michx. (Fabaceae: Papilionoideae), well known for about 30 natural hybrids within the genus^45^,^46^,^47^, offers an ideal system for studying hybridization, introgression, and incomplete lineage sorting. *Lespedeza* belongs to tribe Desmodieae subtribe Lespedezinae. It comprises 45 herbaceous and shrubby species dispersed throughout East Asia and eastern North America (44 spp. in Ohashi and Nemoto^48^; one additional sp. in Xu *et al*.)^49^. *Lespedeza* is traditionally divided into two subgenera, i.e., subg. *Lespedeza* and subg. *Macrolespedeza* (Maxim.) H. Ohashi^50^,^51^,^52^. As traditionally defined, species of subg. *Macrolespedeza* are shrubs with only chasmogamous flowers while those of subg. *Lespedeza* are herbs or subshrubs with both cleistogamous and chasmogamous flowers. Geographically, species of subg. *Lespedeza* occur disjunctly in eastern North America and East Asia, while those of subg. *Macrolespedeza* are limited to East Asia. The two-subgenus classification based on macromorphology is supported by pollen features^53^. However, neither of the subgenera is recovered as monophyletic by a study based on one nuclear and five plastid markers; instead, the American species and Asian species of *Lespedeza* each were resolved as monophyletic and sister to each other^51^. Most recently, Ohashi and Nemoto^48^ proposed a new infrageneric system which confined subg. *Lespedeza* to North America, and subg. *Macrolespedeza* to Asia mainly based on the results of recent molecular work^51^.

Based on ITS and five plastid markers, Xu *et al*.^51^ found that the nuclear and plastid markers contained obviously incongruent phylogenetic signals at shallow-level phylogeny. Also, the resolution among species within the traditionally defined subg. *Macrolespedeza* was very poor. Surprisingly, multiple samples of the same species within subg. *Macrolespedeza* from different localities often did not group together based on either ITS or plastid markers. As we have known recently, polymorphic ITS copies within individuals have been identified in many different plant groups which may confound attempts to recover correct phylogenetic species relationships. Our preliminary study indicated that there is relatively high nrDNA ITS diversity within individuals of some species of *Lespedeza*, implying incomplete concerted evolution. Additionally, the uniparental inheritance of plastid gene tree reflects evolutionary processes other than those of phylogenetic descent which may mask evidence of reticulation in hybridizing species^54^,^55^. Either of the reasons may cause incongruence between the ITS and plastid phylogenies.

The objectives of this study included: (1) to thoroughly investigate the extent and pattern of intra- and inter-specific ITS diversity in *Lespedeza*; (2) to distinguish the presence and pattern of pseudogenes; (3) to reconstruct the reticulate evolution of species of *Lespedeza*; and (4) to compare newly obtained evolutionary relationships with complete distribution pattern of ITS clones to understand the impact of hybridization and introgression on the evolution of the ITS lineages and on phylogenetic reconstruction.

## Results

A total of 555 sequences were newly generated for this study (Supplementary Information: Appendix).

### Length variation and sequence diversity of ITS region

In total, 450 ITS clones were obtained from the 50 samples of *Lespedeza*. Two hundred ninety out of 450 clones were distinct clones. The entire length of ITS region (containing partial 18S and 26S) ranged from 587 (*L. stuevei*-3, including a 136-bp deletion in ITS1 and 5.8S) to 732 bp, and their alignment was 808 bp long, of which 546 (67.6%) were variable and 442 (54.7%) were potentially parsimony informative. The length of ITS1, 5.8S, ITS2 region varied from 127 (*L. stuevei*-3, including a 111-bp deletion) to 246 bp, 104 (*L. patens*-5, including a 59-bp deletion) to 163 bp, and 183 (*L. caraganae*-8, including a 30-bp deletion) to 218 bp, respectively.

### GC content of the ITS region, secondary structure of 5.8S rDNA sequences and identification of pseudogenes and recombinants

As shown in Table S1 (Supplementary Information), the GC content of *Lespedeza* ranged from 46.55% to 67.06% in ITS1, 41.76% to 56.05% in 5.8S, and 46.43% to 69.48% in ITS2 region. Within the clones, 35 ITS sequences had significantly lower GC contents in at least one of three regions compared with that of others from the same sample. Most of the clones exhibited the predicted secondary structure for a functional 5.8S rRNA, with the presence of five conserved helices. However, 28 clones displayed non-compensatory substitutions and at least one helix could not be folded correctly. Thirty clones contained at least one substitution within one of the three conserved motifs. The nucleotide changes in three conserved motives of 5.8S rDNA accessions are summarized in Table 1. Based on the information of sequence length and substitution variation, GC content, presence of conserved motives in the 5.8S rDNA sequence, and ability of 5.8S rDNA sequence to fold into a conserved secondary structure, we identified 37 putative pseudogenes (Table 1). The sequence diversity across the ITS region of all putative pseudogenes, estimated either by the number of nucleotide differences (k) or by nucleotide diversity (Pi), was remarkably higher than the presumed functional sequences (Table 2).

Five putative pseudogenes (*Lepedeza bicolor*-1-6, *L. jiangxiensis*-2-5, *L. lichiyuniae*-3-8, *L. patens*-10, and *L. wilfordii*-1-7) were detected as recombinants with the RDP4 program. Another two recombinants (*L. jiangxiensis*-1-2, *L. jiangxiensis*-2-8) were identified by their sharp discontinuities in the patterns of sequence similarity and substitution pattern, though RDP4 did not identify these.

### ITS phylogenetic analyses

**Figure 1:**
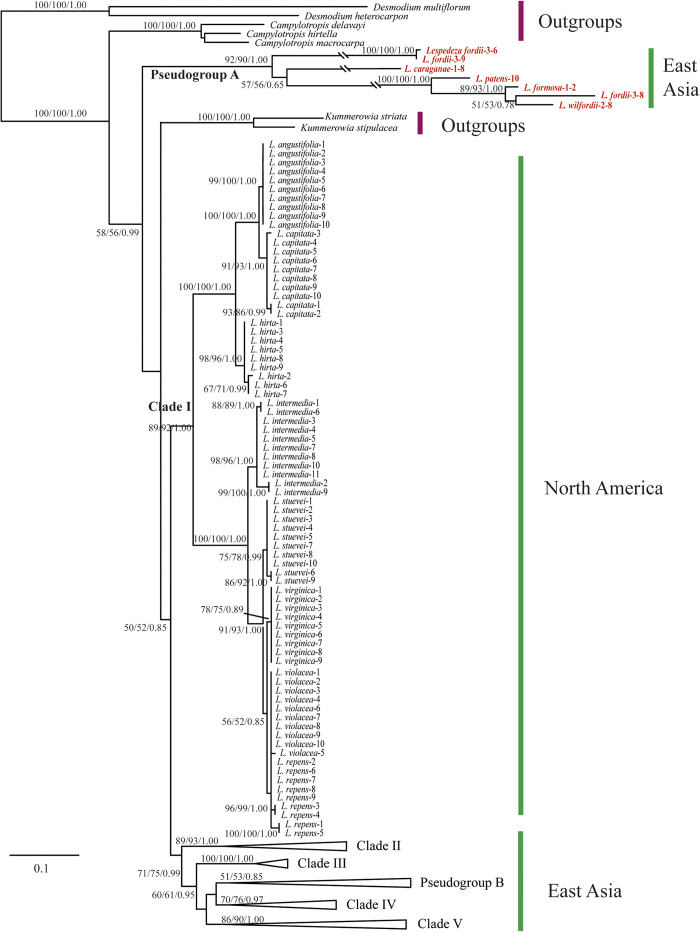
Phylogram resulting from Maximum likelihood phylogenetic analysis of ITS cloning data. There are seven main clades within *Lespedeza* (Clades I, II, III, IV, V, and Pseudogroups (**A,B**).They are expanded in Figs 2, 3, 4. Support values (maximum likelihood bootstrap support, maximum parsimony jackknife support and posterior probability) are shown along the branches. Dash shows that the bootstrap value or jackknife value is lower than 50. Pseudogenes are marked in red.

**Figure 2:**
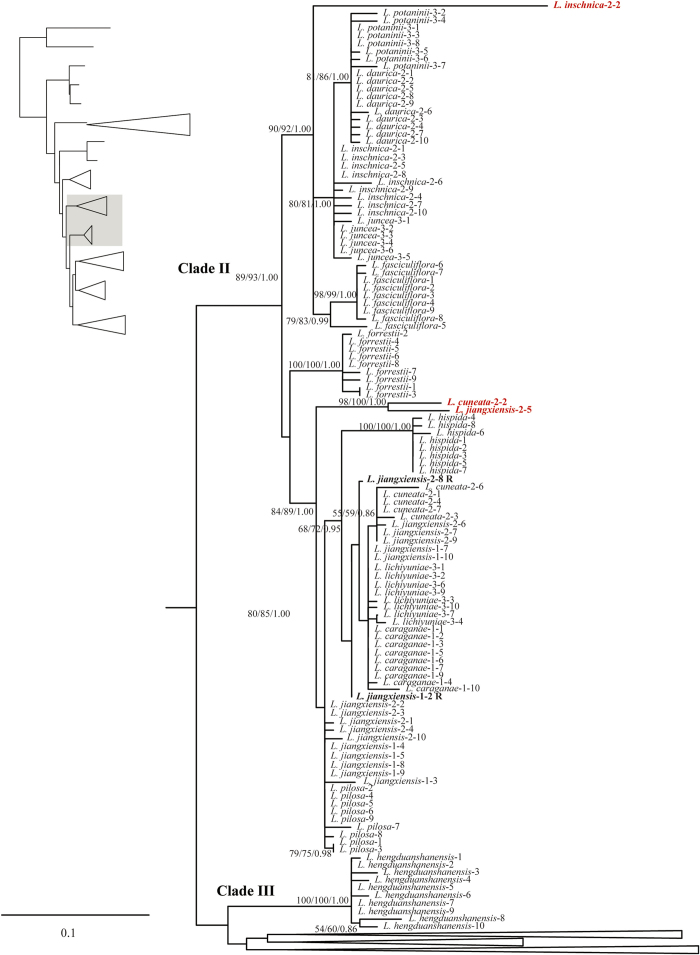
Detail of Clades II, III from Maximum likelihood phylogenetic analysis of ITS cloning data. Support values (maximum likelihood bootstrap support, maximum parsimony jackknife support and posterior probability) are shown along the branches. Dash shows that the bootstrap value or jackknife value is lower than 50. Pseudogenes are marked in red and recombinants are labeled R.

**Figure 3:**
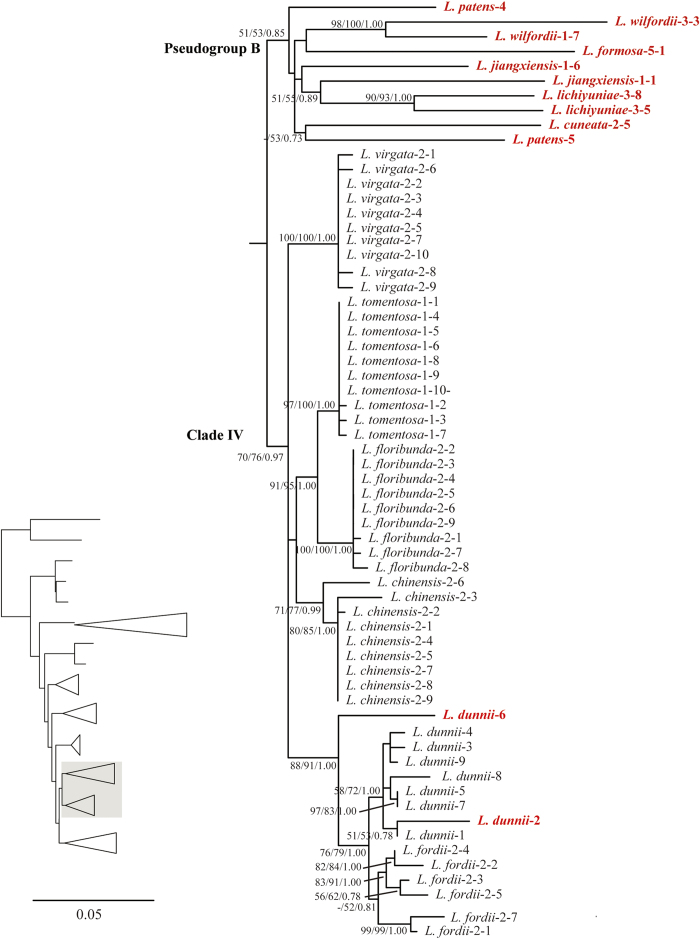
Detail of Clade IV, Pseudogroup B from Maximum likelihood phylogenetic analysis of ITS cloning data. Support values (maximum likelihood bootstrap support, maximum parsimony jackknife support and posterior probability) are shown along the branches. Dash shows that the bootstrap value or jackknife value is lower than 50. Pseudogenes are marked in red.

**Figure 4:**
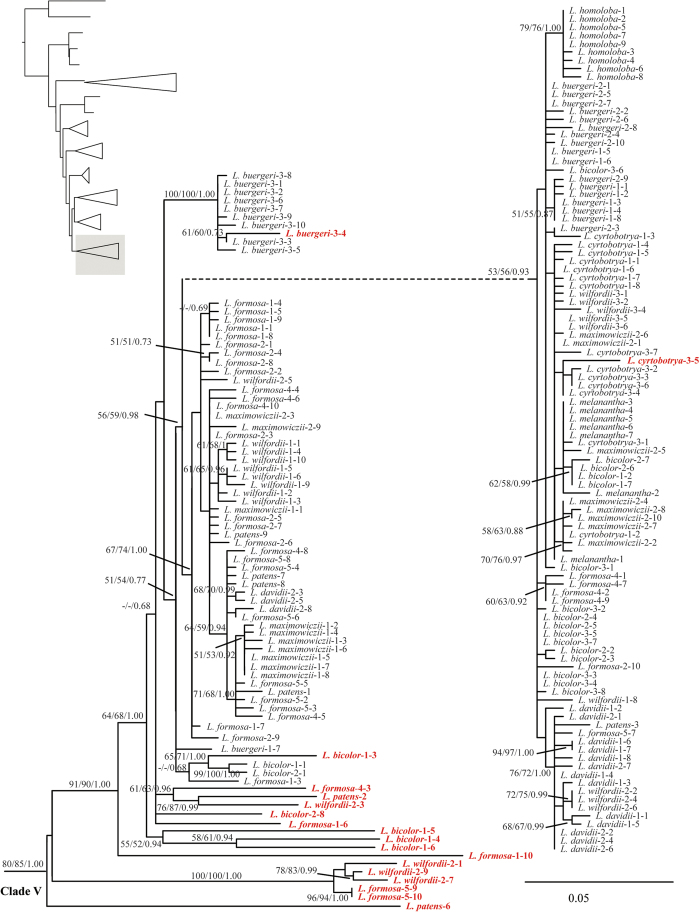
Detail of Clade V from Maximum likelihood phylogenetic analysis of ITS cloning data. Support values (maximum likelihood bootstrap support, maximum parsimony jackknife support and posterior probability) are shown along the branches. Dash shows that the bootstrap value or jackknife value is lower than 50. Pseudogenes are marked in red.

The ML analysis resulted in one optimal tree which is shown in Figs 1, 2, 3, 4, with ML bootstrap (LB), MP jackknife support values (PJ), and Bayesian posterior probabilities (PP). The MP and Bayesian tree topologies were similar (no well-supported conflicts) to the results from the ML analysis. Both *Campylotropis* and *Kummerowia* were strongly supported as monophyletic (LB = 100%, PJ = 100%, PP = 1.00), whereas *Lespedeza* was not resolved as monophyletic by influence of the putative pseudogenes. Within *Lespedeza*, seven major clades were identified (Fig. 1) which consisted of two pure pseudogene clades (Pseudogroups A, B) and five main functional gene clades (Clades I, II, III, IV, V). The relationships among Pseudogroup A, *Kummerowia*, and the rest of *Lespedeza* were unresolved. North American species of *Lespedeza* formed a well supported monophyletic group (Fig. 1: Clade I; LB = 89%, PJ = 92%, PP = 1.00) without any putative pseudogene, which was sister to the Asian species of *Lespedeza* except Pseudogroup A. Within the Asian species, six major clades were recovered and most of the relationships among them were unresolved (Fig. 1: Clades II, III, IV, V, Pseudogroups A, B).

### PGK phylogenetic analyses

The final PGK dataset contained 105 accessions and 1,494 aligned nucleotides, of which 358 (24.0%) were variable and 198 (13.3%) were parsimony informative.

**Figure 5:**
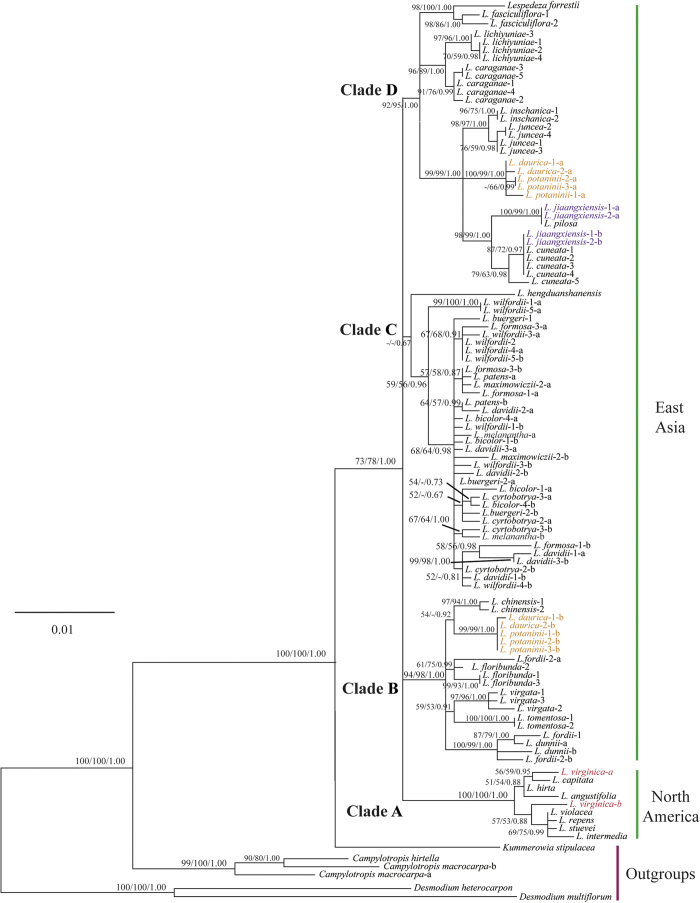
Phylogram resulting from Maximum likelihood phylogenetic analysis of PGK data. There are four main clades within *Lespedeza* (Clades **A,B,C,D**). Support values (maximum likelihood bootstrap support, maximum parsimony jackknife support and posterior probability) are shown along the branches. Dash shows that the bootstrap value or jackknife value is lower than 50. Typical hybrid species are marked in multicolor.

The phylogenetic analyses of the PGK dataset using MP, ML, and BI methods showed similar topologies. *Lespedeza* was resolved as a monophyletic group with moderate to strong support (LB = 73%, PJ = 78%, PP = 1.00) and is sister to *Kummerowia* and these two together are further sister to *Campylotropis*. Within *Lespedeza*, four major clades were identified (Fig. 5: Clades A, B, C, D). Three of the them were well supported (LB ≥ 92%, PJ = ≥ 95%, PP = 1.00), while one was weakly supported (PP = 0.67). North American species formed a well supported monophyletic group and further divided into two monophyletic subgroups with weak support (Fig. 5: Clade A). Within the Asian species, three major clades were recovered and their relationships were unresolved (Fig. 5: Clades B, C, D). Surprisingly, many samples show two divergent copies within an individual, e.g., *L. daurica* (Laxm.) Schindl., *L. jiangxiensis* Bo Xu, X.F.Gao & Li Bing Zhang, *L. potaninii* Vassiliev, and most samples of Clade C.

## Discussion

### Characterization of ITS pseudogenes and recombinants in *Lespedeza*

In recent years, divergent ITS paralogs which may contain non-functional pseudogenes have been observed in more and more plant groups^13^,^16^,^17^,^18^,^19^,^20^,^21^,^22^,^23^. ITS pseudogenes can be identified with the GC content, secondary structure stability, substitution rates, the presence of conserved motifs, and phylogenetic positions^6^,^19^,^21^,^22^. In the present study, most ITS pseudogenes could be easily detected according to nucleotide changes in conserved 5.8S motives, the significantly lower GC contents in at least one of three regions, and the lost ability of 5.8S rDNA sequence to fold into a conserved secondary structure. As expected, all ITS pseudogenes in *Lespedeza* accumulated methylation related substitutions, which reduced the GC content and led to the loss of gene function. In agreement with other studies^19^, the putative pseudogenes in *Lespedeza* showed remarkably increased nucleotide substitutions and sequence diversity compared with presumed functional sequences.

Our phylogenetic results suggested that the putative pseudogenes in *Lespedeza* are mainly divided into three groups, i.e., Pseudogroup A (LB = 92%, PJ = 90%, PP = 1.00), Pseudogroup B (LB = 51%, PJ = 53%, PP = 0.85), and some others are mixed with functional copies. Pseudogroups A and B formed a large genetic differentiation with functional copies, suggesting that these pseudogenes originated with a long history. There are three different distribution patterns for the mixed pseudogenes: 1) The differentiation between pseudogenes and their functional copies is relatively small and they are still resolved in a monophyletic clade in the phylogenetic analysis, e.g., *L. buergeri*-3-4, *L. cyrtobotrya*-3-5 (Fig. 4), suggesting that their origins did not predate the divergence of the species; 2) The differentiation between pseudogenes and their functional copies is relatively large and the pseudogenes are resolved in a separate clade, e.g., *L. inschnica*-2-2, *L. formosa*-1-10 (Figs 2 and 4); and 3) The pseudogenes from different species form a well-supported clade probably due to the result of long-branch attraction, e.g., *L. cuneata*-2-2, *L. jiangxiensis*-2-5 (Fig. 2).

For the recombinants in *Lespedeza* detected by the RDP4 program, most of their putative parent sequences were also found to be pseudogenes. The putative recombination mainly occur between non-functional pseudogenes maybe due to their early origin and accumulation of high-level homoplastic mutations. The two recombinants (*L. jiangxiensis*-1-2, *L. jiangxiensis*-2-8; Fig. 2) identified by their sharp discontinuities are resolved in basal positions to either parental lineage, which indicates that they may have resulted in the loss of clade resolution in the phylogenetic tree and obscure the real phylogenetic relationships. However, we could not distinguish the recombinants by PCR-mediated recombination from those by natural recombination.

### Incomplete concerted evolution of ITS in *Lespedeza*

Despite the concerted evolution of rDNA, polymorphic ITS copies within individuals have been identified in many different plant groups^10^,^16^,^18^,^19^,^20^,^23^. The high-level intra-individual polymorphism (290 out of 446) in the ITS region in *Lespedeza* suggests that incomplete concert evolution exists in this genus. Divergent paralogs of ITS sequences, including putative pseudogenes, multiple functional ITS copies, and probably also recombinants, were detected in the same individual in our study.

Interestingly, the resolution patterns of the putative functional clones were highly different between the two traditional subgenera. Within subg. *Macrolespedeza*, the clones from most species were mixed together, suggesting low rate of concerted evolution in this subgenus. In contrast, clones from each species of subg. *Lespedeza* each formed a monophyletic group or unresolved, implying a higher rate of concerted evolution in the subgenus. The distinct resolution patterns of the ITS clones in the two subgenera can be attributed to their different evolutionary histories. Several biological processes might retard or disrupt concert evolution, such as polyploidization^56^,^57^, agamospermy^58^, multiple nucleolar organizer regions (NORs;^24^,^57^,^59^,^60^, longer generation times^61^, and hybridization^17^,^20^,^25^,^58^. In *Lespedeza*, all taxa investigated so far except *L. daurica* and *L. potaninii* are diploid^62^,^63^,^64^ and no agamospermous taxa have been reported. The number of NORs in *Lespedeza* is currently unknown, but most diploid angiosperms have only one or two NORs per genome^38^,^65^,^66^. Thus, polyploidization, agamospermy, and a large number of NORs may not be the major reasons for the intra-individual polymorphism and great difference of resolution patterns of the ITS clones in the two subgenera. A long generation time has been suggested as a mechanism that might retard concerted evolution in *Paeonia*^61^. Within *Lespedeza*, members of subg. *Lespedeza* are herbs or subshrubs, while those of subg. *Macrolespedeza* are shrubs. Although we do not have any detailed information on the generation times of the two subgenera, normally shrubs have relatively longer generation times than herbs and subshrubs. Therefore, different generation time may have been one of the reasons responsible for the different resolution patterns of the ITS clones in the two subgenera. Hybridization could be accounted for the high-level intra-individual polymorphism of ITS found in many other plant groups, including *Amelanchier* Medik^58^, *Iris* L.^67^, Musaceae^20^, *Paeonia* L.^61^, *Platanus* L.^17^, *Pyrus* L.^18^, and *Quercus* L.^25^. Since interspecific hybridization is well-known within *Lespedeza*^45^,^46^,^47^, it appears to be plausible to assume that hybridization may have created the similar high-level intra-individual polymorphism of ITS in the genus. The great difference between the resolution patterns of the ITS clones in the two subgenera could be attributed to their different extents/frequencies of hybridization. This inference is further corroborated by the analysis of nuclear gene PGK which shows rampant hybridization may have played a key role in the evolution of subg. *Macrolespedeza*.

### Phylogeny of *Lespedeza*

In our present phylogenetic analyses based on ITS paralogs, *Lespedeza* is not resolved as monophyletic (Fig. 1) because of the putative pseudogenes, whereas it is resolved as a monophyletic group with better support based on PGK gene data than it was in our previous study based on ITS and plastid loci^51^. North American species are strongly supported as monophyletic based on PGK gene data, in accordance with previous studies. In contrast, the monophyly of the Asian taxa is not recovered based on PGK gene data but resolved as paraphyletic in relation to the North American species and *Kummerowia*, different from previous studies which resolved the Asian species as monophyletic and sister to the North American species^51^,^52^. The PGK data do not support Ohashi & Nemoto^48^ classification which recognized the Asian and North American species as two subgenera. Notably, monophyletic groups are generally recognized, while some authors advocated for paraphyletic groups.

The major clades in *Lespedeza* identified with previous ITS and plastid data are recovered in the analyses of PGK data (Fig. 5: Clades A, B, C, D). However, PGK data are found to provide better resolution among the species complexes, especially within Clade D (Fig. 5). For example, the relationships among *L. caraganae* Bunge, *L. cuneata* (Dum. Cours.) G. Don, *L. inschanica* Schindl., *L. juncea* Pers., and *L. lichiyuniae* Nemoto & Ohashi were unresolved based on previous ITS and plastid data^51^, but are well resolved based on the PGK data. In the PGK gene tree (Fig. 5), multiple accessions contain two divergent copies within an individual, e.g., *L. daurica, L. jiangxiensis, L. potaninii*, and most accessions of Clade C, which is suggestive that frequent hybridization may have played an important role in the evolution of *Lespedeza*.

### Hybridization within *Lespedeza*

Natural hybridization is recognized as an important creative force and evolutionary process in generating angiosperm species diversity^26^,^27^,^68^. The signature of hybridization can be revealed by phylogenetic incongruence between different markers, or the existence of two divergent alleles of single-copy nuclear gene within one individual. Our new data, as well as previous study^51^, suggest that several species or species complexes of *Lespedeza* may be of hybrid origin and hybridization may have played a critical role in the evolution of the genus. Hybrid speciation within *Lespedeza* occurs either at a homoploid level (between two species of the same ploidy) or via allopolyploidy (speciation via hybridization and genome doubling). Listed below are several examples of the hybridization within *Lespedeza*.

*Lespedeza jiangxiensis* was newly described from Jiangxi Province, China^69^. Our previous study^51^ based on ITS and five plastid markers identified *L. pilosa* Siebold & Zucc. as the closest relative of *L. jiangxiensis*. Interestingly, however, *L. pilosa* is remarkably different from *L. jiangxiensis* morphologically. In the present study, we have identified two divergent PGK copies in *L. jiangxiensis* (Fig. 5: Clade D). The two divergent copies grouped with *L. cuneata* and *L. pilosa*, respectively, indicating that *L. jiangxiensis* is a hybrid progeny formed between *L. cuneata* and *L. pilosa*. Furthermore, *L. pilosa* can be inferred as the female parent when analyzed in concert with plastid markers^51^.

*Lespedeza virginica* (L.) Britton is a North American species with purple flowers. In the earlier studies based on both ITS and plastid markers^52^,^70^, *L. virginica* formed a clade with *L. intermedia* (S. Watson ex A. Gray) Britton, *L. procumbens* Michx., *L. repens* (L.) W.P.C. Barton, *L. stuevei* Nutt., and *L. violacea* (L.) Pers., which all have purple flowers. In our previous study^51^, the position of *L. virginica* in the ITS tree was in accordance with that in earlier studies, but in our tree based on combined plastid data *L. virginica* grouped with *L. angustifolia* (Pursh) Elliott, *L. capitata* Michx., *L. hirta* (L.) Hornem., and *L. leptostachya* Engelm. ex A. Gray, which all have white flowers. This suggests that the plastids of our *L. virginica* sample are acquired from another taxon with white flowers (e.g., *L. angustifolia, L. capitata, L. hirta*, or *L. leptostachya*). This hypothesis is corroborated by our new nuclear PGK data in this study, which clearly indicate that our *L. virginica* sample is a hybrid progeny formed between *L. virginica* and *L. capitata* (Fig. 5: Clade A).

*Lespedeza daurica* and *L. potaninii* are only tetraploid species known so far in the genus^62^,^63^,^71^. The relationships of *L. daurica* and *L. potaninii* have been well discussed. Some taxonomists regarded *L. potaninii* as a subspecies of *L. daurica* or a species of its own^50^,^72^. However, Ohashi *et al*.^73^ considered *L. potaninii* being conspecific with *L. daurica* and treated *L. potaninii* as an ecological form growing in sunny dry areas. Our previous study^51^ showed that *L. daurica* and *L. potaninii* formed a monophyletic clade and were closely related with *L. inschanica* and *L. juncea*. In the present study (Fig. 5), all five accessions of *L. daurica* and *L. potaninii* each have two divergent copies and these two copies each formed a monophyletic clade (Fig. 5: Clades B, D), suggesting that *L. daurica* and *L. potaninii* originated from interbreeding between *L. chinensis* (weak support) and a species of Clade D (e.g., *L. cuneata, L. inschanica, L. juncea*). This hypothesis is reinforced by chromosome evidence. The chromosome number 2n = 22 is common throughout Clade B and 2n = 20 throughout Clade D, while the chromosome numbers of *L. daurica* and *L. potaninii* are 2n = 42^62^,^63^,^71^. Therefore, the chromosomal data well support the speciation of *L. daurica* and *L. potaninii* via hybridization between Clade B and Clade D resulting in genome doubling.

Subg. *Macrolespedeza* is characterized by having a woody habit and only chasmogamous flowers in compound racemes. In our previous study based on ITS and plastid data^51^, multiple accessions of the same species of subg. *Macrolespedeza* from different geographical localities often did not group together. It appears that multiple copies of some genes exist intensively in subg. *Macrolespedeza*. Surprisingly, this hypothesis is corroborated by our present study which shows that all the twenty samples of subg. *Macrolespedeza* except *L. buergeri*-1 and *L. wilfordii*-2 have two divergent PGK copies (Fig. 5: Clade C). Two reasons can be accounted for an individual having two copies of a single-copy nuclear gene: (1) It has undergone a gene duplication for this gene; and (2) It is a hybrid progeny while the two copies are different alleles from different parents. In the first case, if there is a gene duplication of PGK gene in the evolution of subg. *Macrolespedeza*, the divergent copies of the subgenus should be grouped into two distinct clades. However, in our study, the divergent copies from most species are mixed together on the tree (Fig. 5). Thus, gene duplication can not be the major factor for the remarkable presence of such divergent PGK gene copies in subg. *Macrolespedeza*. Notably, species of subg. *Macrolespedeza* are often geographically distributed sympatrically and have not yet been clearly distinguished from one another due to their lack of significant morphological characters and the presence of interspecific hybrids^46^,^51^,^64^. The two divergent copies from most species were mixed together on the tree, implying that widespread and frequent hybridization may have played a significant role in the evolution of subg. *Macrolespedeza*. This is consistent with the findings of previous studies on other groups of plants, which showed that hybridization is particularly likely to occur in plant species complexes in which species are evolutionarily young and sometimes not well differentiated^32^,^33^,^34^,^35^,^36^,^37^.

### Parents homogenization of ITS

Our putative hybrid samples of *L. daurica, L. potaninii*, and *L. virginica*, based on PGK gene and plastid loci which only showed one parental repeat type in the analysis of ITS clones, indicating that the alternative repeat type may be lost due to the impact of concerted evolution which may have caused parents homogenization. In *L. virginica*, homogenization of polymorphism is biased towards paternal type copies. By contrast, in allopolyploid species of *L. daurica* and *L. potaninii*, all the samples show a biased homogenization towards maternal type repeats. Different directions, degrees, and patterns of concerted evolution have been reported in different plant groups especially among allopolyploids^6^,^38^,^41^,^74^,^75^. For example, in *Cardamine* L. (Brassicaceae), homogenization towards maternal alleles were uniform across multiple accessions of allopolyploid species^74^. Similarly, in most populations of both tetraploids, *Tragopogon* L. (Poaceae) showed a reduction in paternal rDNA homologs^76^. In contrast, in *Nicotiana tabacum* L. (Solanaceae), after hybridization of the parental diploid species, the paternal dominated its rDNA and the maternal rDNA repeat was then eliminated from the allopolyploid genome^77^. Meanwhile, bidirectional interlocus concerted evolution, in which repeats become homogenized to alternative progenitor diploids in different allopolyploid species, was also found in *Gossypium* L.^38^,^75^ and *Oryza* L.^38^,^75^. Despite the underlying driven mechanisms responsible for different unidirectional homogenization are not well understood in our study, the parents-biased homogenization of ITS following hybridization and polyploidization has very significant implications for phylogeny reconstruction, especially when based on rDNA sequences. If not well recognized, such biased homogenization following hybridization or polyploidization could lead to incorrect tree inference and erroneous estimates of the evolutionary relationships.

### The impact of ITS evolution on phylogeny

The ITS region, as a spacer diverging at relatively high rate, is one of the most widely used molecular makers in phylogenetic inference of plants at generic and species levels. Since intra-individual polymorphisms and pseudogenes of ITS were discovered in *Zea* L.^11^, they have been increasingly detected in many plant groups^15^,^16^,^17^,^18^,^19^,^20^,^21^,^23^,^25^,^58^. Many of these studies show and discuss the phylogenetic consequences of the existence of pseudogenes. Zheng *et al*.^18^ found that functional ITS copies led to poorly resolved phylogeny as a result of low sequence divergence, while certain types of pseudogenes and some relict pseudogenes offered more credible clues for the evolutionary history of species of *Pyrus* (Rosaceae). Similar results have been obtained by Razafimandimbison *et al*.^15^ in Naucleeae (Rubiaceae), which clearly showed that divergent putative pseudogenes could be useful for phylogenetic analyses, especially when no sequences of their functional counterparts were available. Although the two examples above demonstrate that pseudogenes can be helpful to resolve phylogenetic relationships of closely related species to a certain extent, in most cases, non-concerted ITS evolution due to the existence of pseudogenes brings many difficulties, and even leads to misunderstanding of evolutionary relationships among taxa^10^,^16^,^17^,^19^,^23^,^25^,^78^,^79^. In our present study, divergent pseudogenes in *Lespedeza* accumulated high-level homoplastic mutations leading to random relationships among species partially due to long-branch attractions. Similar results were found in Brassicaceae and *Cycas* L.^13^,^19^. Recombination of divergent sequences following hybridization leads to chimeric DNA sequences which may also cause phylogenetic errors. Two recombinations of ITS (might have been resulted from PCR process though) are discovered in our putative hybrid species *L. jiangxiensis* and are resolved as basal to the parental lineages. Alvarez and Wendel^6^ reviewed the recombination of divergent sequences following hybridization in several different plants and found that this process may mask the real phylogenetic signals. As mentioned above, the parents-biased homogenization of ITS following hybridization and polyploidization can also result in incorrect tree inference and erroneous estimates of the evolutionary relationships. Therefore, when using ITS marker for reconstructing phylogeny and studying evolution of hybridization and polyploidization, one must be aware of parents homogenization and the impact of pseudogenes and recombinations.

## Methods

### Taxon sampling

In total, 50 samples representing 37 species of *Lespedeza* were cloned for nrDNA ITS sequence analyses and 71 samples representing 36 species of *Lespedeza* were included for PGK gene analyses. We also included two species of *Kummerowia* Schindl. [*K. stipulacea* (Maximowicz) Makino and *K. striata* (Thunberg) Schindl.], three species of *Campylotropis* Bunge [*C. delavayi* (Franch.) Schindl., *C. hirtella* (Franch.) Schindl., and *C. macrocarpa* (Bunge) Rehd.], and two species of *Desmodium* Desv. [*D. microphyllum* (Thunb.) DC. and *D. heterocarpon* (Linn.) DC.] as outgroups, following Xu *et al*.^51^. Most of our samples were collected in the field and in a few cases leaf material was directly taken from herbarium specimens. Voucher information and GenBank accession numbers for each accession are listed in Appendix.

### DNA extraction, amplification, and sequencing

Total genomic DNA was extracted from silica-dried material or sometimes from herbarium fragments using the TIANGEN plant genomic DNA extraction kit (TIANGEN Biotech., Beijing, China) following the manufacturers’ protocols.

The internal transcribed spacer (ITS) and a single-copy nuclear gene coding for phosphoglycerate kinase (PGK) were used in this study. The ITS sequences were amplified with primers ITS4 and ITSA^80^. Those of PGK region were amplified with newly designed primers xb_pgk1F (GACAGTATT GGTCCAGAAGTAG) and xb_pgk1R (CAGAATCTCCTCCTCCAATAAT).

All PCR conditions followed Xu *et al*.^51^. Amplified fragments were purified with the TIANquick Mini Purification Kit (TIANGEN). Purified PCR products were sequenced by Invitrogen (Shanghai, China). If sequences had more than one heterozygous peak or could not be directly sequenced in PGK, cloning was performed. All the accessions of ITS within *Lespedeza* were cloned. Cloning was carried out using the pEASY-T3 Cloning Kit (TransGen Biotech, Beijing, China), following the manufacturer’s protocols. Finally, six to twelve positive clones were sequenced.

### Sequence treatment, alignment and gap coding

Sequencher 4.1 (Gene Codes Corp., Ann Arbor, MI, USA) was used to assemble and edit complementary strands. PCR errors were corrected by comparing the cloned sequences with the original sequences from direct sequencing. Sequences obtained for each fragment were initially aligned using Clustal X 1.81^81^ and then manually adjusted in BioEdit^82^. Gap characters were coded following the modified complex indel coding^83^,^84^ for maximum parsimony (MP) and simple coding^84^ for the Bayesian Inference (BI) using SeqState^85^. Single-base repeats as well as ambiguously aligned indels were excluded.

### ITS sequence analysis, secondary structure reconstruction, and recombination test

The boundaries of the ITS1, 5.8S and ITS2 regions were identified according to previously published ITS sequences from GenBank. GC content calculation and haplotype identification were performed using MEGA4^86^. DnaSP^87^ was used to calculate the average number of nucleotide difference (K), the nucleotide diversity (p). Secondary structures of the 5.8S region were predicted under specific settings for base pairing (Hribova *et al*.^20^, for helix B4, F 45 105 3; helix B5, F 48 61 3; helix B6, F 69 96 3; helix B7, F 110 118 3; and for helix B8, F119 142 4 and F 126 135 3) using Mfold version 2.3 on a web server (http://unafold.rna.albany.edu/? q=mfold/RNA-Folding-Form2.3)^88^. For the 5.8S rDNA sequences, we inspected for the presence of three angiosperm conserved motifs: M1(5′-CGATGAAGAACGTAGC), M2 (5′-GAATTGCAGAATCC-3′), and M3 (5′-TTTGAACGCA-3′) following Harpke and Peterson^89^. Recombination Detection Program (RDP4)^90^ was used to detect putative recombinants.

### Phylogenetic analysis

Unweighted maximum parsimony (MP) analyses were conducted for each locus in PAUP* ver. 4.0b10^91^ using 1000 tree-bisection-reconnection (TBR) searches with MAXTREES set to increase without limit. Parsimony jackknife (JK) analyses^92^ with heuristic search were conducted using PAUP* with the removal probability set to approximately 37%, and “jac” resampling emulated. One thousand replicates were performed with 10 TBR searches per replicate.

jModelTest v2.1.1^93^ was used to select the best-fitting likelihood model for each dataset. The best-fitting models and parameter values selected using the Akaike Information Criterion^94^.

Maximum likelihood (ML)^95^ tree searches and ML bootstrapping were performed using RAxML-HPC2 on XSEDE v8.0.24^96^ on the web server Cipres Science Gateway^97^, with 5000 rapid bootstrap analyses followed by a search for the best-scoring tree in a single run.

Bayesian inference (BI) was conducted with mixed models using MrBayes v3.2.1^98^. Two runs of four Markov chain Monte Carlo chains were conducted, each beginning with a random tree and sampling one tree every 1000 generations of 10,000,000 generations. Convergence among chains was checked using Tracer 1.5^99^ and the first 25% was discarded as burn-in. The remaining trees were used to calculate a 50% majority-rule consensus topology and posterior probabilities (PP).

## Additional Information

**How to cite this article**: Xu, B. *et al*. ITS non-concerted evolution and rampant hybridization in the legume genus *Lespedeza* (Fabaceae). *Sci. Rep.* **7**, 40057; doi: 10.1038/srep40057 (2017).

**Publisher's note:** Springer Nature remains neutral with regard to jurisdictional claims in published maps and institutional affiliations.

## Supplementary Material

Supplementary Information

## Figures and Tables

**Table 1:** Sequence characteristics of putative ITS pseudogenes in *Lespedeza.*

Accession name	GC content [%]			Position of nucleotide changes (nt-) in conserved 5.8S motives			Secondary structure of 5.8S	Note
	ITS1	5.8S	ITS2	M1	M2	M3		
*L. bicolor-*1-3	**62.30**	55.41	67.14	**nt-16**	conserved	conserved	XXXXX	pseudogene
*L. bicolor-*1-4	**63.52**	**50.96**	**63.85**	**nt-16**	conserved	conserved	XXXXX	pseudogene
*L. bicolor-*1-5	**61.89**	53.50	**62.19**	**nt-12**	conserved	**nt-6**	**X-XX-**	pseudogene
*L. bicolor-*1-6	**62.30**	**50.96**	**63.38**	**nt-16**	conserved	conserved	XXXXX	pseudogene
*L. bicolor-*2-8	64.75	55.41	**64.73**	**nt-4**	conserved	conserved	**X-XXX**	pseudogene
*L. buergeri*-3-4	65.16	52.87	67.61	**nt-16**	conserved	**nt-8**	**XXX--**	pseudogene
*L. caraganae*-8	**52.23**	**45.86**	**52.46**	**nt-12**	**nt-7,12**	**nt-7,9**	**X**-**X**--	pseudogene
*L. cuneata-*2-2	65.59	54.78	**64.79**	conserved	conserved	conserved	XXXXX	pseudogene
*L. cuneata-*2-5	61.18	52.87	**61.03**	**nt-6**	**nt-8**	**nt-8**	**XXX**--	pseudogene
*L. cyrtobotrya*-3-5	**63.34**	55.41	68.54	conserved	conserved	**nt-8**	XXXXX	pseudogene
*L. dunnii-*2	63.22	55.41	**62.39**	conserved	conserved	conserved	XXXXX	pseudogene
*L. dunnii-*6	**59.49**	55.41	64.79	conserved	**nt-6**	conserved	XXXXX	pseudogene
*L. fordii-*3-6	**52.05**	**47.77**	**51.64**	**nt-16**	**nt-6,7**	**nt-2,7**	-----	pseudogene
*L. fordii-*3-8	**46.55**	**41.76**	**46.43**	**nt-1,12, 16**	**nt-6,14**	**nt-8,9,10**	**X**----	pseudogene
*L. fordii-*3-9	**51.64**	**47.77**	**51.64**	**nt-16**	**nt-6,7**	**nt-2,7**	-----	pseudogene
*L. formosa-*1-2	**47.41**	**42.24**	**47.88**	**nt-9,12, 16**	**nt-6,14**	**nt-8,9,10**	-----	pseudogene
*L. formosa-*1-6	63.11	**52.87**	**63.85**	conserved	**nt-6**	conserved	XXXXX	pseudogene
*L. formosa-*1-10	**60.25**	**50.96**	**61.03**	conserved	conserved	**nt-9**	**XX**---	pseudogene
*L. formosa*-4-3	63.11	53.50	65.10	conserved	**nt-7**	**nt-1**	-**XXXX**	pseudogene
*L. formosa-*5-1	**57.74**	**49.68**	**57.75**	conserved	**nt-7**	**nt-7**	**XX--X**	pseudogene
*L. formosa-*5-9	**58.51**	**51.28**	**59.62**	**nt-14**	conserved	**nt-4**	**XX-X-**	pseudogene
*L. formosa-*5-10	**58.51**	**51.28**	**59.62**	**nt-14**	conserved	**nt-4**	**XX-X-**	pseudogene
*L. inschnica-*2-2	**61.48**	**52.23**	**63.85**	conserved	**nt-7**	**nt-8**	**XXX**-**X**	pseudogene
*L. jiangxiensis-*1-1	**58.61**	**50.96**	**59.62**	**nt-16**	conserved	**conserved**	**XXXX**-	pseudogene
*L. jiangxiensis-*1-6	**58.61**	54.03	66.67	conserved	**nt-1**	**all missing**	**XXX**--	pseudogene
*L. jiangxiensis-*2-5	64.78	54.78	**63.38**	conserved	conserved	conserved	XXXXX	pseudogene
*L. lichiyuniae-*3-5	**60.08**	**51.59**	**58.69**	**nt-16**	**nt-7**	**conserved**	XXXXX	pseudogene
*L. lichiyuniae-*3-8	65.18	54.78	**60.09**	conserved	conserved	conserved	**XXXX**-	pseudogene
*L. patens-*2	**61.63**	54.14	**64.32**	conserved	conserved	conserved	XXXXX	pseudogene
*L. patens-*4	**57.74**	54.14	**61.03**	conserved	conserved	conserved	**XXXX**-	pseudogene
*L. patens-*5	**56.49**	**59.18**	**60.09**	**missing 4-16**	**all missing**	**nt-2**	---**XX**	pseudogene
*L. patens-*6	**58.09**	**49.68**	**57.28**	**nt-16**	conserved	**nt-7**	**XX-XX**	pseudogene
*L. patens-*10	**54.20**	**42.31**	**47.02**	**nt-9,12,16**	**nt-6,14**	**nt-8,9,10**	-----	pseudogene
*L. wilfordii-*1-7	65.73	53.50	**60.56**	conserved	conserved	conserved	**XXXX**-	pseudogene
*L. wilfordii-*2-1	**61.89**	**51.92**	**60.09**	**nt-14**	conserved	**nt-4**	**XXXX**-	pseudogene
*L. wilfordii-*2-3	63.93	54.78	**62.91**	conserved	conserved	conserved	XXXXX	pseudogene
*L. wilfordii-*2-7	**59.43**	**51.92**	**59.62**	**nt-14**	conserved	**nt-4**	**XXXX**-	pseudogene
*L. wilfordii-*2-8	**46.98**	**42.31**	**47.62**	**nt-8,9,12, 16**	**nt-6,14**	**nt-8,9,10**	-----	pseudogene
*L. wilfordii-*2-9	**59.84**	**51.92**	**60.09**	**nt-14**	conserved	**nt-4**	**XXXX**-	pseudogene
*L. wilfordii-*3-3	**54.51**	**51.59**	**59.62**	**nt-2,11**	**nt-9**	conserved	**X**-**XXX**	pseudogene

Indicates the presence (x) or absence (−) of the ability to build up the helices (B4–B8), respectively.

**Table 2:** Nucleotide diversity of individual parts and the entire ITS region of *Lespedeza* analyzed.

Region	ITS1		5.8S		ITS2		ITS	
Sequence type	F	P	F	P	F	P	F	P
Pi	0.067	0.139	0.005	0.154	0.061	0.179	0.038	0.146
k	7.357	23.827	0.473	12.765	10.614	27.376	18.825	57.927

F – presumed functional ITS paralogs; P – putative ITS pseudogenes; Pi – Nucleotide diversity; k – Average number of nucleotide differences.
