# Supplementary material for: ITS non-concerted evolution and rampant hybridization in the legume genus Lespedeza (Fabaceae)
Source: Sci Rep. 2017 Jan 4;7:40057. doi: 10.1038/srep40057 (PMC5209741; doi:10.1038/srep40057)
Supplement: Supplementary Information [file srep40057-s1.pdf]

ITS non-concerted evolution and rampant hybridization in the legume genus *Lespedeza* (Fabaceae)

Bo Xu[1], Xiao-Mao Zeng[1], Xin-Fen Gao[1, *], Dong-Pil Jin[2], Li-Bing Zhang[3, *]

[1]*CAS Key Laboratory of Mountain Ecological Restoration and Bioresource Utilization & Ecological Restoration and Biodiversity Conservation Key Laboratory of Sichuan Province, Chengdu Institute of Biology, Chinese Academy of Sciences, P.O. Box 416, Chengdu, Sichuan 610041, China*

[2]*Department of Biological Sciences, Inha University, Incheon 402-751, Republic of Korea*

[3]*Missouri Botanical Garden, P.O. Box 299, St. Louis, Missouri 63166, U.S.A.*

[*]*Authors for correspondence (xfgao@cib.ac.cn; Libing.Zhang@mobot.org)*

Appendix

List of taxa sampled with information related to taxonomy, voucher information, GenBank accession numbers, and references.

Campylotropis hirtella (Franch.) Schindl., *X. F. Gao, Y. Zhang & Z. M. Zhu 11010* (CDBI), Yunnan, China: ITS JN402491 (Xu et al., 2012), PGK KY174339. ***Campylotropis macrocarpa*** (Bunge) Rehd., *B. Xu & L. B. Zhang 97* (CDBI), Guizhou, China: ITS JN402492 (Xu et al., 2012), PGK (a) KY174340, (b) KY174341.

Desmodium heterocarpon (L.) DC., *B. Xu & L. B. Zhang 40* (CDBI), Guizhou, China: PGK KY174342. ***Desmodium microphyllum*** (Thunb.) DC., *B. Xu 219* (CDBI), Jiangxi, China: PGK KY174343.

Kummerowia stipulacea (Maxim.) Makino, *B. Xu & Z. M. Zhu 370* (CDBI), Liaoning, China: ITS JN402493, PGK KY174344. ***Kummerowia striata*** (Thunb.) Schindl. *N. C. Henderson 04-01* (MO), Alabama, USA: ITS JN402494.

Lespedeza angustifolia (Pursh) Elliott, *R. kral 64371* (MO), Tennessee, USA: ITS (-1) KY174444, (-2) KY174445, (-3) KY174446, (-4) KY174447, (-5) KY174448, (-6) KY174449, (-7) KY174450, (-8) KY174451, (-9) KY174452, (-10) KY174453, PGK KY174345. ***Lespedeza bicolor*** Turcz., **1**, *B. Xu & Z. M. Zhu 379* (CDBI), Liaoning, China: ITS (-1) KY174454, (-2) KY174455, (-3) KY174456, (-4) KY174457, (-5) KY174458, (-6) KY174459, (-7) KY174460, PGK (a) KY174346, (b) KY174347; **2**, *B. Xu 248* (CDBI), Zhejiang, China: ITS (-1) KY174461, (-2) KY174462, (-3) KY174463, (-4) KY174464, (-5) KY174465, (-6) KY174466, (-7) KY174467, (-8) KY174468; **3**, *B. Xu & Z. M. Zhu 356* (CDBI), Hebei, China: ITS (-1) KY174469, (-2) KY174470, (-3) KY174471, (-4) KY174472, (-5) KY174473, (-6) KY174474, (-7) KY174475, (-8) KY174476; **4**, , *K. Yonekura, 11711* (PE), Hokkaido, Japan: PGK (a) KY174348, (b) KY174349. ***Lespedeza buergeri*** Miq., **1**, *B. Xu & Z. M. Zhu 333-2* (CDBI), Henan, China: ITS (-1) KY174477, (-2) KY174478, (-3) KY174479, (-4) KY174480, (-5) KY174481, (-6) KY174482, (-7) KY174483, (-8) KY174484, PGK KY174350; **2**, *B. Xu*

187 (CDBI), Jiangxi, China: ITS (-1) KY174485, (-2) KY174486, (-3) KY174487, (-4) KY174488, (-5) KY174489, (-6) KY174490, (-7) KY174491, (-8) KY174492, (-9) KY174493, (-10) KY174494, PGK (a) KY174351, (b) KY174352; **3**, *T. Azuma & C. Tanifuji 382* (PE), Honshu, Japan: ITS (-1) KY174495, (-2) KY174496, (-3) KY174497, (-4) KY174498, (-5) KY174499, (-6) KY174500, (-7) KY174501, (-8) KY174502, (-9) KY174503. ***Lespedeza capitata*** Michx., *N. C. Henderson 02-97* (MO), Missouri, USA: ITS (-1) KY174504, (-2) KY174505, (-3) KY174506, (-4) KY174507, (-5) KY174508, (-6) KY174509, (-7) KY174510, (-8) KY174511, (-9) KY174512, (-10) KY174513, PGK KY174353. ***Lespedeza caraganae*** Bunge, **1**, *B. Xu & Z. M. Zhu 353* (CDBI), Hebei, China: ITS (-1) KY174514, (-2) KY174515, (-3) KY174516, (-4) KY174517, (-5) KY174518, (-6) KY174519, (-7) KY174520, (-8) KY174521, (-9) KY174522, (-10) KY174523, PGK KY174354; **2**, *B. Xu & Z. M. Zhu 384* (CDBI), Beijing, China: PGK KY174355; **3**, *B. Xu & Z. M. Zhu 326-1* (CDBI), Henan, China: PGK KY174356; **4**, *B. Xu & Z. M. Zhu 326-15* (CDBI), Henan, China: PGK KY174357; **5**, *B. Xu & Z. M. Zhu 326-25* (CDBI), Henan, China: PGK KY174358. ***Lespedeza chinensis*** G. Don, **1**, *B. Xu 202* (CDBI), Jiangxi, China: PGK KY174359; **2**, *B. Xu 227* (CDBI), Jiangxi, China: ITS (-1) KY174524, (-2) KY174525, (-3) KY174526, (-4) KY174527, (-5) KY174528, (-6) KY174529, (-7) KY174530, (-8) KY174531, (-9) KY174532, (-10) KY174533, PGK KY174360. ***Lespedeza cuneata*** (Dum. Cours.) G. Don, **1**, *B. Xu & L. B. Zhang 88-1* (CDBI), Guizhou, China: PGK KY174361; **2**, *B. Xu & L. B. Zhang 88-3* (CDBI), Guizhou, China: ITS (-1) KY174534, (-2) KY174535, (-3) KY174536, (-4) KY174537, (-5) KY174538, (-6) KY174539, (-7) KY174540, PGK KY174362; **3**, *B. Xu & L. B. Zhang 90-11* (CDBI), Guizhou, China: PGK KY174363; **4**, *B. Xu & L. B. Zhang 90-19* (CDBI), Guizhou, China: PGK KY174364; **5**, *B. Xu & W. G. Tu 403* (CDBI), Sichuan, China: PGK KY174365. ***Lespedeza cyrtobotrya*** Miq., **1**, *B. Xu & Z. M. Zhu 338* (CDBI), Henan, China: ITS (-1) KY174541, (-2) KY174542, (-3) KY174543, (-4) KY174544, (-5) KY174545, (-6) KY174546, (-7) KY174547, (-8) KY174548; **2**, *B. Xu 151* (CDBI), Shaanxi, China: PGK (a) KY174366, (b) KY174367; **3**, D. P. Jin 138843 (CDBI), Honshu, Japan: ITS (-1) KY174549, (-2) KY174550, (-3) KY174551, (-4) KY174552, (-5) KY174553, (-6) KY174554, (-7) KY174555, PGK (a) KY174368, (b) KY174369. ***Lespedeza daurica*** (Laxm.) Schindl., **1**, *B. Xu & Z. M. Zhu 369-13* (CDBI), Liaoning, China: PGK (a) KY174370, (b) KY174371; **2**, *B. Xu 126* (CDBI), Shaanxi, China: ITS (-1) KY174556, (-2) KY174557, (-3) KY174558, (-4) KY174559, (-5) KY174560, (-6) KY174561, (-7) KY174562, (-8) KY174563, (-9) KY174564, (-10) KY174565, PGK (a) KY174372, (b) KY174373. ***Lespedeza davidii*** Franch., **1**, *B. Xu & L. B. Zhang 98* (CDBI), Guizhou, China: ITS (-1) KY174566, (-2) KY174567, (-3) KY174568, (-4) KY174569, (-5) KY174570, (-6) KY174571, (-7) KY174572, (-8) KY174573, PGK (a) KY174374, (b) KY174375; **2**, *B. Xu 191* (CDBI), Jiangxi, China: ITS (-1) KY174574, (-2) KY174575, (-3) KY174576, (-4) KY174577, (-5) KY174578, (-6) KY174579, (-7) KY174580, (-8) KY174581, PGK (a) KY174376, (b) KY174377; **3**, *B.Xu 238* (CDBI), Zhejiang, China: PGK (a) KY174378, (b) KY174379. ***Lespedeza dunnii*** Schindl., B. Xu 255 (CDBI), Zhejiang, China: ITS (-1) KY174582, (-2) KY174583, (-3) KY174584, (-4) KY174585, (-5) KY174586, (-6) KY174587, (-7) KY174588, (-8) KY174589, (-9) KY174590, PGK (a) KY174380, (b) KY174381. ***Lespedeza***

fasciculiflora Franch., **1**, *X. F. Gao & B. Xu 9594* (CDBI), Yunnan, China: ITS (-1) KY174591, (-2) KY174592, (-3) KY174593, (-4) KY174594, (-5) KY174595, (-6) KY174596, (-7) KY174597, (-8) KY174598, (-9) KY174599, PGK KY174382; **2**, *X. F. Gao & B. Xu 9776* (CDBI), Sichuan, China: PGK KY174383. ***Lespedeza floribunda*** Bunge, **1**, *B. Xu & L. B. Zhang 398* (CDBI), Beijing, China: PGK KY174384; **2**, *B. Xu & L. B. Zhang 95-11* (CDBI), Guizhou, China: ITS (-1) KY174600, (-2) KY174601, (-3) KY174602, (-4) KY174603, (-5) KY174604, (-6) KY174605, (-7) KY174606, (-8) KY174607, (-9) KY174608, PGK KY174385; **3**, *B. Xu 127-28* (CDBI), Shaanxi, China: PGK KY174386. ***Lespedeza fordii*** Schindl., **1**, *B. Xu 192* (CDBI), Jiangxi, China: PGK KY174387; **2**, *B. Xu 226* (CDBI), Jiangxi, China: ITS (-1) KY174609, (-2) KY174610, (-3) KY174611, (-4) KY174612, (-5) KY174613, (-6) KY174614, (-7) KY174615, (-8) KY174616, (-9) KY174617, PGK (a) KY174388, (b) KY174389. ***Lespedeza formosa*** (Vogel) Koehne, **1**, *B. Xu & L. B. Zhang 1* (CDBI), Guizhou, China: ITS (-1) KY174618, (-2) KY174619, (-3) KY174620, (-4) KY174621, (-5) KY174622, (-6) KY174623, (-7) KY174624, (-8) KY174625, (-9) KY174626, (-10) KY174627, PGK (a) KY174390, (b) KY174391; **2**, *B. Xu 184-1* (CDBI), Hubei, China: ITS (-1) KY174628, (-2) KY174629, (-3) KY174630, (-4) KY174631, (-5) KY174632, (-6) KY174633, (-7) KY174634, (-8) KY174635, (-9) KY174636, (-10) KY174637; **3**, *B. Xu & Z. M. Zhu 337* (CDBI), Henan, China: PGK (a) KY174392, (b) KY174393; **4**, *B. Xu 195* (CDBI), Jiangxi, China: ITS (-1) KY174638, (-2) KY174639, (-3) KY174640, (-4) KY174641, (-5) KY174642, (-6) KY174643, (-7) KY174644, (-8) KY174645, (-9) KY174646, (-10) KY174647; **5**, *B. Xu 262-12* (CDBI), Zhejiang, China: ITS (-1) KY174648, (-2) KY174649, (-3) KY174650, (-4) KY174651, (-5) KY174652, (-6) KY174653, (-7) KY174654, (-8) KY174655, (-9) KY174656, (-10) KY174657. ***Lespedeza* forrestii** Schindl., *X. F. Gao & B. Xu 9626* (CDBI), Yunnan, China: ITS (-1) KY174658, (-2) KY174659, (-3) KY174660, (-4) KY174661, (-5) KY174662, (-6) KY174663, (-7) KY174664, (-8) KY174665, (-9) KY174666, PGK KY174394. ***Lespedeza hengduanshanensis*** (C. J. Chen) Xu, Gao & Zhang, *X. F. Gao & B. Xu 9384* (CDBI), Sichuan, China: ITS (-1) KY174667, (-2) KY174668, (-3) KY174669, (-4) KY174670, (-5) KY174671, (-6) KY174672, (-7) KY174673, (-8) KY174674, (-9) KY174675, (-10) KY174676, PGK KY174395. ***Lespedeza hirta*** (L.) Hornem., *R. Dale 97963* (MO), Louisiana, USA: ITS (-1) KY174677, (-2) KY174678, (-3) KY174679, (-4) KY174680, (-5) KY174681, (-6) KY174682, (-7) KY174683, (-8) KY174684, (-9) KY174685, PGK KY174396. ***Lespedeza hispida*** (Franch) Nemoto & Ohashi, *X. F. Gao 504* (KUN), Yunnan, China: ITS (-1) KY174686, (-2) KY174687, (-3) KY174688, (-4) KY174689, (-5) KY174690, (-6) KY174691, (-7) KY174692, (-8) KY174693. ***Lespedeza homoloba*** Nakai, *M. Takahashi & K. Takahashi 1613* (PE), Shikoku, Japan: ITS (-1) KY174694, (-2) KY174695, (-3) KY174696, (-4) KY174697, (-5) KY174698, (-6) KY174699, (-7) KY174700, (-8) KY174701, (-9) KY174702. ***Lespedeza inschanica*** Schindl., **1**, *B. Xu & Z. M. Zhu 378* (CDBI), Liaoning, China: PGK KY174397; **2**, *B. Xu 143* (CDBI), Shaanxi, China: ITS (-1) KY174703, (-2) KY174704, (-3) KY174705, (-4) KY174706, (-5) KY174707, (-6) KY174708, (-7) KY174709, (-8) KY174710, (-9) KY174711, (-10) KY174712, PGK KY174398. ***Lespedeza intermedia*** (S. Watson ex A. Gray) Britton, *R. D. Thomas, E. Sundell & C. Amason 107866* (MO), Arkansas, USA: ITS (-1)

KY174713, (-2) KY174714, (-3) KY174715, (-4) KY174716, (-5) KY174717, (-6) KY174718, (-7) KY174719, (-8) KY174720, (-9) KY174721, (-10) KY174722, (-11) KY174723, PGK KY174399. ***Lespedeza jiangxiensis*** Xu, Gao & Zhang, **1**, *B. Xu 198* (CDBI), Jiangxi, China: ITS (-1) KY174724, (-2) KY174725, (-3) KY174726, (-4) KY174727, (-5) KY174728, (-6) KY174729, (-7) KY174730, (-8) KY174731, (-9) KY174732, (-10) KY174733, PGK (a) KY174400, (b) KY174401; **2**, *B. Xu 218* (CDBI), Jiangxi, China: ITS (-1) KY174734, (-2) KY174735, (-3) KY174736, (-4) KY174737, (-5) KY174738, (-6) KY174739, (-7) KY174740, (-8) KY174741, (-9) KY174742, (-10) KY174743, PGK (a) KY174402, (b) KY174403. ***Lespedeza juncea*** (L) Pers., **1**, *X. F. Gao 9836* (CDBI), Beijing, China: PGK KY174404; **2**, *B. Xu & Z. M. Zhu 372* (CDBI), Liaoning, China: PGK KY174405; **3**, *B. Xu & Z. M. Zhu 377* (CDBI), Liaoning, China: ITS (-1) KY174744, (-2) KY174745, (-3) KY174746, (-4) KY174747, (-5) KY174748, (-6) KY174749, PGK KY174406; **4**, *B. Xu & Z. M. Zhu 358* (CDBI), Hebei, China: PGK KY174407. ***Lespedeza lichiyuniae*** Nemoto & Ohashi, **1**, *B. Xu & L. B. Zhang 57* (CDBI), Guizhou, China: PGK KY174408; **2**, *B. Xu & L. B. Zhang 47* (CDBI), Guizhou, China: PGK KY174409; **3**, *X. M. An 3641* (PE), Guizhou, China: ITS (-1) KY174750, (-2) KY174751, (-3) KY174752, (-4) KY174753, (-5) KY174754, (-6) KY174755, (-7) KY174756, (-8) KY174757, (-9) KY174758, (-10) KY174759, PGK KY174410; **4**, *B. Xu 433* (PE), Zhejiang, China: PGK KY174411. ***Lespedeza maximowiczii*** C.K. Schneid., **1**, *B. Xu 265* (CDBI), Henan, China: ITS (-1) KY174760, (-2) KY174761, (-3) KY174762, (-4) KY174763, (-5) KY174764, (-6) KY174765, (-7) KY174766, (-8) KY174767; **2**, *B. Xu 237* (CDBI), Zhejiang, China: ITS (-1) KY174768, (-2) KY174769, (-3) KY174770, (-4) KY174771, (-5) KY174772, (-6) KY174773, (-7) KY174774, (-8) KY174775, (-9) KY174776, (-10) KY174777, PGK (a) KY174412, (b) KY174413. ***Lespedeza melanantha*** Nakai, *D. P. Jin 128151* (CDBI), Kyeongsangnamdo, Korea: ITS (-1) KY174778, (-2) KY174779, (-3) KY174780, (-4) KY174781, (-5) KY174782, (-6) KY174783, (-7) KY174784, PGK (a) KY174414, (b) KY174415. ***Lespedeza patens*** Nakai, *B. Xu 233* (CDBI), Jiangxi, China: ITS (-1) KY174785, (-2) KY174786, (-3) KY174787, (-4) KY174788, (-5) KY174789, (-6) KY174790, (-7) KY174791, (-8) KY174792, (-9) KY174793, (-10) KY174794, PGK (a) KY174416, (b) KY174417. ***Lespedeza pilosa*** Siebold & Zucc., *B. Xu 257* (CDBI), Zhejiang, China: ITS (-1) KY174795, (-2) KY174796, (-3) KY174797, (-4) KY174798, (-5) KY174799, (-6) KY174800, (-7) KY174801, (-8) KY174802, (-9) KY174803, PGK KY174418. ***Lespedeza potaninii*** Vassiliev, **1**, *B. Xu 272* (CDBI), Shaanxi, China: PGK (a) KY174419, (b) KY174420; **2**, *B. Xu 404* (CDBI), Sichuan, China: PGK (a) KY174421, (b) KY174422; **3**, *B. Xu 9684* (CDBI), Sichuan, China: ITS (-1) KY174804, (-2) KY174805, (-3) KY174806, (-4) KY174807, (-5) KY174808, (-6) KY174809, (-7) KY174810, (-8) KY174811, PGK (a) KY174423, (b) KY174424. ***Lespedeza repens*** (L.) W. P. C. Barton, *N. C. Henderson 95-683* (MO), Kansas, USA: ITS (-1) KY174812, (-2) KY174813, (-3) KY174814, (-4) KY174815, (-5) KY174816, (-6) KY174817, (-7) KY174818, (-8) KY174819, (-9) KY174820, PGK KY174425. ***Lespedeza stuevei*** Nutt., *C. Becker 1444* (MO), Missouri, USA: ITS (-1) KY174821, (-2) KY174822, (-3) KY174823, (-4) KY174824, (-5) KY174825, (-6) KY174826, (-7) KY174827, (-8) KY174828, (-9) KY174829, (-10) KY174830, PGK KY174426. ***Lespedeza tomentosa***

(Thunb.) Siebold ex Maxim., **1**, *B. Xu & L. B. Zhang 55* (CDBI), Guizhou, China: ITS (-1) KY174831, (-2) KY174832, (-3) KY174833, (-4) KY174834, (-5) KY174835, (-6) KY174836, (-7) KY174837, (-8) KY174838, (-9) KY174839, (-10) KY174840, PGK KY174427; **2**, *B. Xu 145* (CDBI), Shaanxi, China: PGK KY174428. ***Lespedeza violacea*** (L.) Pers., *L. B. Zhang 4817* (MO), Missouri, USA: ITS (-1) KY174841, (-2) KY174842, (-3) KY174843, (-4) KY174844, (-5) KY174845, (-6) KY174846, (-7) KY174847, (-8) KY174848, (-9) KY174849, (-10) KY174850, PGK KY174429. ***Lespedeza virgata*** (Thunb.) DC., **1**, *B. Xu & L. B. Zhang 104* (CDBI), Guizhou, China: PGK KY174430; **2**, *B. Xu 201* (CDBI), Jiangxi, China: ITS (-1) KY174851, (-2) KY174852, (-3) KY174853, (-4) KY174854, (-5) KY174855, (-6) KY174856, (-7) KY174857, (-8) KY174858, (-9) KY174859, (-10) KY174860, PGK KY174431; **3**, *B. Xu 324* (CDBI), Shaanxi, China: PGK KY174432. ***Lespedeza virginica*** (L.) Britton, *L. B. Zhang 4816* (MO), Missouri, USA: ITS (-1) KY174861, (-2) KY174862, (-3) KY174863, (-4) KY174864, (-5) KY174865, (-6) KY174866, (-7) KY174867, (-8) KY174868, (-9) KY174869, PGK (a) KY174433, (b) KY174434. ***Lespedeza wilfordii*** Ricker, **1**, *B. Xu 206-1* (CDBI), Jiangxi, China: ITS (-1) KY174870, (-2) KY174871, (-3) KY174872, (-4) KY174873, (-5) KY174874, (-6) KY174875, (-7) KY174876, (-8) KY174877, (-9) KY174878, (-10) KY174879, PGK (a) KY174435, (b) KY174436; **2**, *B.Xu 209* (CDBI), Jiangxi, China: ITS (-1) KY174880, (-2) KY174881, (-3) KY174882, (-4) KY174883, (-5) KY174884, (-6) KY174885, (-7) KY174886, (-8) KY174887, (-9) KY174888, PGK KY174437; **3**, *B. Xu 252* (CDBI), Zhejiang, China: ITS (-1) KY174889, (-2) KY174890, (-3) KY174891, (-4) KY174892, (-5) KY174893, (-6) KY174894, (-7) KY174895, PGK (a) KY174438, (b) KY174439; **4**, *B. Xu 256* (CDBI), Zhejiang, China: PGK (a) KY174440, (b) KY174441; **5**, *B. Xu 249* (CDBI), Zhejiang, China: PGK (a) KY174442, (b) KY174443.

Table S1. Sequence characteristics of ITS1-5.8S-ITS2 region in *Lespedeza*

Accession name	GC content [%]			Position of nucleotide changes (nt-) in conserved 5.8S motives			Secondary structure of 5.8S	Note
	ITS1	5.8S	ITS2	M1	M2	M3		
L. angustifolia-1	61.48	54.78	62.04	conserved	conserved	conserved	XXXXX	
L .angustifolia-2	61.48	54.78	62.04	conserved	conserved	conserved	XXXXX	
L. angustifolia-3	61.48	54.78	62.04	conserved	conserved	conserved	XXXXX	
L. angustifolia-4	61.48	54.78	62.04	conserved	conserved	conserved	XXXXX	
L. angustifolia-5	61.48	54.78	62.04	conserved	conserved	conserved	XXXXX	
L. angustifolia-6	61.48	54.78	62.04	conserved	conserved	conserved	XXXXX	
L. angustifolia-7	61.48	54.78	62.04	conserved	conserved	conserved	XXXXX	
L. angustifolia-8	61.48	54.78	62.04	conserved	conserved	conserved	XXXXX	
L. angustifolia-9	61.48	54.78	62.04	conserved	conserved	conserved	XXXXX	
L. angustifolia-10	61.48	54.78	62.04	conserved	conserved	conserved	XXXXX	
L. bicolor-1-1	65.45	54.78	67.61	conserved	conserved	conserved	XXXXX	
L. bicolor-1-2	66.40	54.78	68.08	conserved	conserved	conserved	XXXXX	
***L. bicolor*-1-3**	**62.30**	55.41	67.14	**nt-16**	conserved	conserved	XXXXX	**pseudogene**
***L. bicolor*-1-4**	**63.52**	**50.96**	**63.85**	**nt-16**	conserved	conserved	XXXXX	**pseudogene**
***L. bicolor*-1-5**	**61.89**	53.50	**62.19**	**nt-12**	conserved	**nt-6**	**X-XX-**	**pseudogene**
***L. bicolor*-1-6**	**62.30**	**50.96**	**63.38**	**nt-16**	conserved	conserved	XXXXX	**pseudogene**
L. bicolor-1-7	66.40	54.78	68.08	conserved	conserved	conserved	XXXXX	
L. bicolor-2-1	66.26	54.78	67.61	conserved	conserved	conserved	XXXXX	
L. bicolor-2-2	66.27	54.78	68.08	conserved	conserved	conserved	XXXXX	
L. bicolor-2-3	64.02	54.78	67.61	conserved	conserved	conserved	XXXXX	
L. bicolor-2-4	65.87	54.78	67.61	conserved	conserved	conserved	XXXXX	
L. bicolor-2-5	65.87	54.78	67.61	conserved	conserved	conserved	XXXXX	
L. bicolor-2-6	66.40	54.78	68.08	conserved	conserved	conserved	XXXXX	
L. bicolor-2-7	66.80	54.78	68.54	conserved	conserved	conserved	XXXXX	
***L. bicolor*-2-8**	64.75	55.41	**64.73**	**nt-4**	conserved	conserved	**X-XXX**	**pseudogene**
L. bicolor-3-1	66.27	54.78	67.61	conserved	conserved	conserved	XXXXX	
L. bicolor-3-2	65.87	54.78	67.14	conserved	conserved	conserved	XXXXX	
L. bicolor-3-3	65.87	54.78	67.61	conserved	conserved	conserved	XXXXX	
L. bicolor-3-4	65.87	54.78	67.61	conserved	conserved	conserved	XXXXX	
L. bicolor-3-5	65.87	54.78	67.61	conserved	conserved	conserved	XXXXX	
L. bicolor-3-6	65.60	54.78	67.61	conserved	conserved	conserved	XXXXX	
L. bicolor-3-7	65.87	54.78	67.61	conserved	conserved	conserved	XXXXX	
L. bicolor-3-8	65.48	54.78	67.61	conserved	conserved	conserved	XXXXX	
L. buergeri-1-1	66.00	54.78	67.45	conserved	conserved	conserved	XXXXX	
L. buergeri-1-2	66.00	54.78	67.45	conserved	conserved	conserved	XXXXX	
L. buergeri-1-3	66.40	54.78	67.45	conserved	conserved	conserved	XXXXX	
L. buergeri-1-4	66.40	54.78	67.45	conserved	conserved	conserved	XXXXX	
L. buergeri-1-5	66.00	54.78	67.61	conserved	conserved	conserved	XXXXX	
L. buergeri-1-6	66.00	54.78	67.45	conserved	conserved	conserved	XXXXX	
L. buergeri-1-7	64.90	54.78	67.45	conserved	conserved	conserved	XXXXX	
L. buergeri-1-8	66.40	54.78	67.61	conserved	conserved	conserved	XXXXX	
L. buergeri-2-1	66.00	54.78	67.61	conserved	conserved	conserved	XXXXX	
L. buergeri-2-2	66.00	55.41	67.14	conserved	conserved	conserved	XXXXX	
L. buergeri-2-3	66.00	55.41	67.61	conserved	conserved	conserved	XXXXX	
L. buergeri-2-4	65.60	54.78	67.61	conserved	conserved	conserved	XXXXX	
L. buergeri-2-5	66.00	54.78	67.61	conserved	conserved	conserved	XXXXX	

L. buergeri-2-6	65.60	54.14	67.61	conserved	conserved	conserved	XXXXX	
L. buergeri-2-7	66.00	54.78	67.61	conserved	conserved	conserved	XXXXX	
L. buergeri-2-8	66.00	54.14	67.61	conserved	conserved	conserved	XXXXX	
L. buergeri-2-9	66.40	54.78	68.08	conserved	conserved	conserved	XXXXX	
L. buergeri-2-10	66.40	54.78	67.45	conserved	conserved	conserved	XXXXX	
L. buergeri-3-1	65.57	54.78	68.08	conserved	conserved	conserved	XXXXX	
L. buergeri-3-2	65.57	54.78	68.08	conserved	conserved	conserved	XXXXX	
L. buergeri-3-3	65.57	55.41	68.54	conserved	conserved	conserved	XXXXX	
***L. buergeri*-3-4**	65.16	52.87	67.61	**nt-16**	conserved	**nt-8**	**XXX--**	**pseudogene**
L. buergeri-3-5	65.57	54.78	69.01	conserved	conserved	conserved	XXXXX	
L. buergeri-3-6	65.57	54.78	68.08	conserved	conserved	conserved	XXXXX	
L. buergeri-3-7	65.57	54.78	68.08	conserved	conserved	conserved	XXXXX	
L. buergeri-3-8	65.57	54.78	68.54	conserved	conserved	conserved	XXXXX	
L. buergeri-3-9	65.98	54.78	68.54	conserved	conserved	conserved	XXXXX	
L. buergeri-3-10	65.98	54.78	68.08	conserved	conserved	conserved	XXXXX	
L. capitata-1	61.22	54.78	62.50	conserved	conserved	conserved	XXXXX	
L. capitata-2	61.22	54.78	62.04	conserved	conserved	conserved	XXXXX	
L. capitata-3	62.04	54.78	61.57	conserved	conserved	conserved	XXXXX	
L. capitata-4	61.22	54.14	62.50	conserved	conserved	conserved	XXXXX	
L. capitata-5	61.63	54.78	61.57	conserved	conserved	conserved	XXXXX	
L. capitata-6	61.63	54.78	62.04	conserved	conserved	conserved	XXXXX	
L. capitata-7	61.63	54.78	62.04	conserved	conserved	conserved	XXXXX	
L. capitata-8	62.04	54.78	62.04	conserved	conserved	conserved	XXXXX	
L. capitata-9	61.63	54.78	62.04	conserved	conserved	conserved	XXXXX	
L. capitata-10	61.63	54.78	62.50	conserved	conserved	conserved	XXXXX	
L. caraganae-1-1	65.18	54.78	68.08	conserved	conserved	conserved	XXXXX	
L. caraganae-1-2	65.18	54.78	68.08	conserved	conserved	conserved	XXXXX	
L. caraganae-1-3	65.18	54.78	68.08	conserved	conserved	conserved	XXXXX	
L. caraganae-1-4	65.18	54.78	68.08	conserved	conserved	conserved	XXXXX	
L. caraganae-1-5	65.18	54.78	68.08	conserved	conserved	conserved	XXXXX	
L. caraganae-1-6	65.18	54.78	68.08	conserved	conserved	conserved	XXXXX	
L. caraganae-1-7	64.90	54.78	68.08	conserved	conserved	conserved	XXXXX	
***L. caraganae*-1-8**	**52.23**	**45.86**	**52.46**	**nt-12**	**nt-7,12**	**nt-7,9**	**X-X--**	**pseudogene**
L. caraganae-1-9	65.18	54.78	68.08	conserved	conserved	conserved	XXXXX	
L. caraganae-1-10	63.56	54.78	68.08	conserved	conserved	conserved	XXXXX	
L. chinensis-2-1	63.67	55.41	63.85	conserved	conserved	conserved	XXXXX	
L. chinensis-2-2	63.67	55.41	63.85	conserved	conserved	conserved	XXXXX	
L. chinensis-2-3	60.82	55.41	63.85	conserved	conserved	conserved	XXXXX	
L. chinensis-2-4	63.67	55.41	63.85	conserved	conserved	conserved	XXXXX	
L. chinensis-2-5	63.67	55.41	63.85	conserved	conserved	conserved	XXXXX	
L. chinensis-2-6	63.27	55.41	67.14	conserved	conserved	conserved	XXXXX	
L. chinensis-2-7	63.67	55.41	63.85	conserved	conserved	conserved	XXXXX	
L. chinensis-2-8	63.67	55.41	63.85	conserved	conserved	conserved	XXXXX	
L. chinensis-2-9	63.67	55.41	63.85	conserved	conserved	conserved	XXXXX	
L. cuneata-2-1	65.18	54.78	68.54	conserved	conserved	conserved	XXXXX	
***L. cuneata*-2-2**	65.59	54.78	**64.79**	conserved	conserved	conserved	XXXXX	**pseudogene**
L. cuneata-2-3	65.18	54.78	68.08	conserved	conserved	conserved	XXXXX	
L. cuneata-2-4	65.18	54.78	68.54	conserved	conserved	conserved	XXXXX	
***L. cuneata*-2-5**	61.18	52.87	**61.03**	**nt-6**	**nt-8**	**nt-8**	**XXX--**	**pseudogene**
L. cuneata-2-6	63.14	54.78	68.54	conserved	conserved	conserved	XXXXX	
L. cuneata-2-7	64.78	54.78	68.54	conserved	conserved	conserved	XXXXX	

L. cyrtobotrya-1-1	66.27	54.78	68.08	conserved	conserved	conserved	XXXXX	
L. cyrtobotrya-1-2	66.27	54.78	68.08	conserved	conserved	conserved	XXXXX	
L. cyrtobotrya-1-3	65.87	55.41	67.61	conserved	conserved	conserved	XXXXX	
L. cyrtobotrya-1-4	65.87	54.78	68.08	conserved	conserved	conserved	XXXXX	
L. cyrtobotrya-1-5	65.87	54.78	68.54	conserved	conserved	conserved	XXXXX	
L. cyrtobotrya-1-6	65.87	54.78	68.08	conserved	conserved	conserved	XXXXX	
L. cyrtobotrya-1-7	65.87	54.14	68.08	conserved	conserved	conserved	XXXXX	
L. cyrtobotrya-1-8	66.53	54.78	68.08	conserved	conserved	conserved	XXXXX	
L. cyrtobotrya-3-1	66.00	54.78	68.08	conserved	conserved	conserved	XXXXX	
L. cyrtobotrya-3-2	66.80	54.78	68.08	conserved	conserved	conserved	XXXXX	
L. cyrtobotrya-3-3	66.80	54.78	68.08	conserved	conserved	conserved	XXXXX	
L. cyrtobotrya-3-4	66.40	54.78	68.08	conserved	conserved	conserved	XXXXX	
***L. cyrtobotrya*-3-5**	**63.34**	55.41	68.54	conserved	conserved	**nt-8**	XXXXX	**pseudogene**
L. cyrtobotrya-3-6	66.80	54.78	68.08	conserved	conserved	conserved	XXXXX	
L. cyrtobotrya-3-7	65.20	54.78	68.08	conserved	conserved	conserved	XXXXX	
L. daurica-2-1	63.71	54.78	67.61	conserved	conserved	conserved	XXXXX	
L. daurica-2-2	64.49	54.78	67.61	conserved	conserved	conserved	XXXXX	
L. daurica-2-3	63.71	55.41	67.61	conserved	conserved	conserved	XXXXX	
L. daurica-2-4	63.71	55.41	67.61	conserved	conserved	conserved	XXXXX	
L. daurica-2-5	63.71	54.78	67.61	conserved	conserved	conserved	XXXXX	
L. daurica-2-6	63.71	54.14	67.14	conserved	conserved	conserved	XXXXX	
L. daurica-2-7	63.31	54.78	67.61	conserved	conserved	conserved	XXXXX	
L. daurica-2-8	63.71	54.78	67.61	conserved	conserved	conserved	XXXXX	
L. daurica-2-9	63.71	54.78	67.61	conserved	conserved	conserved	XXXXX	
L. daurica-2-10	63.31	54.78	67.61	conserved	conserved	conserved	XXXXX	
L. davidii-1-1	66.12	54.78	68.08	conserved	conserved	conserved	XXXXX	
L. davidii-1-2	65.87	54.78	67.61	conserved	conserved	conserved	XXXXX	
L. davidii-1-3	65.71	54.78	68.08	conserved	conserved	conserved	XXXXX	
L. davidii-1-4	65.71	54.78	68.08	conserved	conserved	conserved	XXXXX	
L. davidii-1-5	66.12	54.78	68.08	conserved	conserved	conserved	XXXXX	
L. davidii-1-6	64.90	54.78	68.08	conserved	conserved	conserved	XXXXX	
L. davidii-1-7	64.90	54.78	68.08	conserved	conserved	conserved	XXXXX	
L. davidii-1-8	66.12	54.78	67.61	conserved	conserved	conserved	XXXXX	
L. davidii-2-1	65.59	54.78	68.54	conserved	conserved	conserved	XXXXX	
L. davidii-2-2	65.59	54.78	68.08	conserved	conserved	conserved	XXXXX	
L. davidii-2-3	65.59	54.78	68.08	conserved	conserved	conserved	XXXXX	
L. davidii-2-4	65.59	54.78	68.08	conserved	conserved	conserved	XXXXX	
L. davidii-2-5	65.59	54.78	68.08	conserved	conserved	conserved	XXXXX	
L. davidii-2-6	65.59	54.78	68.08	conserved	conserved	conserved	XXXXX	
L. davidii-2-7	65.31	54.78	68.08	conserved	conserved	conserved	XXXXX	
L. davidii-2-8	65.20	54.78	68.54	conserved	conserved	conserved	XXXXX	
L. dunnii-1	63.22	55.41	66.20	conserved	conserved	conserved	XXXXX	
***L. dunnii*-2**	63.22	55.41	**62.39**	conserved	conserved	conserved	XXXXX	**pseudogene**
L. dunnii-3	62.40	55.41	66.20	conserved	conserved	conserved	XXXXX	
L. dunnii-4	62.81	55.41	66.20	conserved	conserved	conserved	XXXXX	
L. dunnii-5	62.81	55.41	65.73	conserved	conserved	conserved	XXXXX	
***L. dunnii*-6**	**59.49**	55.41	64.79	conserved	**nt-6**	conserved	XXXXX	**pseudogene**
L. dunnii-7	62.81	55.41	65.73	conserved	conserved	conserved	XXXXX	
L. dunnii-8	62.81	56.05	64.79	conserved	conserved	conserved	XXXXX	
L. dunnii-9	62.81	55.41	66.67	conserved	conserved	conserved	XXXXX	
L. fasciculiflora-1	65.71	54.78	67.61	conserved	conserved	conserved	XXXXX	

L. fasciculiflora-2	65.71	54.78	67.61	conserved	conserved	conserved	XXXXX	
L. fasciculiflora-3	65.71	54.78	67.61	conserved	conserved	conserved	XXXXX	
L. fasciculiflora-4	65.71	54.78	67.61	conserved	conserved	conserved	XXXXX	
L. fasciculiflora-5	65.71	54.78	63.85	conserved	conserved	conserved	XXXXX	
L. fasciculiflora-6	66.12	54.78	67.61	conserved	conserved	conserved	XXXXX	
L. fasciculiflora-7	65.71	54.14	67.61	conserved	conserved	conserved	XXXXX	
L. fasciculiflora-8	65.71	53.80	67.61	conserved	conserved	conserved	XXXXX	
L. fasciculiflora-9	65.71	54.78	67.61	conserved	conserved	conserved	XXXXX	
L. floribunda-2-1	63.18	55.41	65.14	conserved	conserved	conserved	XXXXX	
L. floribunda-2-2	63.18	55.41	64.68	conserved	conserved	conserved	XXXXX	
L. floribunda-2-3	63.18	55.41	64.68	conserved	conserved	conserved	XXXXX	
L. floribunda-2-4	63.18	55.41	64.68	conserved	conserved	conserved	XXXXX	
L. floribunda-2-5	63.18	55.41	64.68	conserved	conserved	conserved	XXXXX	
L. floribunda-2-6	63.18	55.41	64.38	conserved	conserved	conserved	XXXXX	
L. floribunda-2-7	63.18	55.41	65.14	conserved	conserved	conserved	XXXXX	
L. floribunda-2-8	63.18	55.41	64.68	conserved	conserved	conserved	XXXXX	
L. floribunda-2-9	63.18	55.41	64.68	conserved	conserved	conserved	XXXXX	
L. fordii-2-1	62.45	54.78	66.05	conserved	conserved	conserved	XXXXX	
L. fordii-2-2	65.45	54.78	65.12	conserved	conserved	conserved	XXXXX	
L. fordii-2-3	65.45	56.05	66.05	conserved	conserved	conserved	XXXXX	
L. fordii-2-4	65.31	55.41	65.58	conserved	conserved	conserved	XXXXX	
L. fordii-2-5	65.85	55.41	66.05	conserved	conserved	conserved	XXXXX	
***L. fordii*-2-6**	**52.05**	**47.77**	**51.64**	**nt-16**	**nt-6,7**	**nt-2,7**	**-----**	**pseudogene**
L. fordii-2-7	63.45	54.78	64.79	conserved	conserved	conserved	XXXXX	
***L. fordii*-2-8**	**46.55**	**41.76**	**46.43**	**nt-1,12, 16**	**nt-6,14**	**nt-8,9,10**	**X----**	**pseudogene**
***L. fordii*-2-9**	**51.64**	**47.77**	**51.64**	**nt-16**	**nt-6,7**	**nt-2,7**	**-----**	**pseudogene**
L. formosa-1-1	64.92	54.78	68.08	conserved	conserved	conserved	XXXXX	
***L. formosa*-1-2**	**47.41**	**41.24**	**47.88**	**nt-9,12, 16**	**nt-6,14**	**nt-8,9,10**	**-----**	**pseudogene**
L. formosa-1-3	64.75	54.14	67.61	conserved	conserved	conserved	XXXXX	
L. formosa-1-4	64.92	54.78	68.08	conserved	conserved	conserved	XXXXX	
L. formosa-1-5	64.92	54.78	67.61	conserved	conserved		XXXXX	
***L. formosa*-1-6**	63.11	**52.87**	**63.85**	conserved	**nt-6**	conserved	XXXXX	**pseudogene**
L. formosa-1-7	65.73	54.78	67.61	conserved	conserved	conserved	XXXXX	
L. formosa-1-8	64.92	54.78	68.08	conserved	conserved	conserved	XXXXX	
L. formosa-1-9	64.92	54.78	68.08	conserved	conserved	conserved	XXXXX	
***L. formosa*-1-10**	**60.25**	**50.96**	**61.03**	conserved	conserved	**nt-9**	**XX---**	**pseudogene**
L. formosa-2-1	64.92	54.78	68.08	conserved	conserved	conserved	XXXXX	
L. formosa-2-2	65.32	54.78	68.08	conserved	conserved	conserved	XXXXX	
L. formosa-2-3	64.92	54.78	68.08	conserved	conserved	conserved	XXXXX	
L. formosa-2-4	65.73	54.78	68.54	conserved	conserved	conserved	XXXXX	
L. formosa-2-5	64.92	54.78	67.61	conserved	conserved	conserved	XXXXX	
L. formosa-2-6	63.31	54.14	68.08	conserved	conserved	conserved	XXXXX	
L. formosa-2-7	64.92	54.78	68.08	conserved	conserved	conserved	XXXXX	
L. formosa-2-8	65.73	54.78	68.08	conserved	conserved	conserved	XXXXX	
L. formosa-2-9	64.92	54.14	67.61	conserved	conserved	conserved	XXXXX	
L. formosa-2-10	65.32	53.50	66.67	conserved	conserved	conserved	XXXXX	
L. formosa-4-1	65.87	54.78	68.08	conserved	conserved	conserved	XXXXX	
L. formosa-4-2	66.27	54.78	67.61	conserved	conserved	conserved	XXXXX	
***L. formosa*-4-3**	63.11	53.50	65.10	conserved	**nt-7**	**nt-1**	**-XXXX**	**pseudogene**
L. formosa-4-4	64.14	54.78	67.14	conserved	conserved	conserved	XXXXX	
L. formosa-4-5	66.13	54.78	68.08	conserved	conserved	conserved	XXXXX	

L. formosa-4-6	64.94	54.78	67.61	conserved	conserved	conserved	XXXXX	
L. formosa-4-7	67.06	54.78	67.61	conserved	conserved	conserved	XXXXX	
L. formosa-4-8	64.92	55.41	68.08	conserved	conserved	conserved	XXXXX	
L. formosa-4-9	66.27	54.78	67.61	conserved	conserved	conserved	XXXXX	
L. formosa-4-10	64.54	54.78	68.08	conserved	conserved	conserved	XXXXX	
***L. formosa*-5-1**	**57.74**	**49.68**	**57.75**	conserved	**nt-7**	**nt-7**	**XX--X**	**pseudogene**
L. formosa-5-2	65.46	54.78	68.08	conserved	conserved	conserved	XXXXX	
L. formosa-5-3	65.86	54.14	67.61	conserved	conserved	conserved	XXXXX	
L. formosa-5-4	64.92	54.78	68.08	conserved	conserved	conserved	XXXXX	
L. formosa-5-5	65.86	54.78	67.61	conserved	conserved	conserved	XXXXX	
L. formosa-5-6	65.32	54.78	68.54	conserved	conserved	conserved	XXXXX	
L. formosa-5-7	65.32	54.78	67.61	conserved	conserved	conserved	XXXXX	
L. formosa-5-8	65.32	54.78	68.08	conserved	conserved	conserved	XXXXX	
***L. formosa*-5-9**	**58.51**	**51.28**	**59.62**	**nt-14**	conserved	**nt-4**	**XX-X-**	**pseudogene**
***L. formosa*-5-10**	**58.51**	**51.28**	**59.62**	**nt-14**	conserved	**nt-4**	**XX-X-**	**pseudogene**
L. forrestii-1	66.26	54.78	66.05	conserved	conserved	conserved	XXXXX	
L. forrestii-2	65.85	54.78	66.51	conserved	conserved	conserved	XXXXX	
L. forrestii-3	66.26	54.78	66.05	conserved	conserved	conserved	XXXXX	
L. forrestii-4	65.85	54.78	66.05	conserved	conserved	conserved	XXXXX	
L. forrestii-5	66.85	54.78	66.05	conserved	conserved	conserved	XXXXX	
L. forrestii-6	65.85	54.78	66.05	conserved	conserved	conserved	XXXXX	
L. forrestii-7	66.67	54.78	66.05	conserved	conserved	conserved	XXXXX	
L. forrestii-8	65.85	54.78	66.05	conserved	conserved	conserved	XXXXX	
L. forrestii-9	66.26	54.78	66.05	conserved	conserved	conserved	XXXXX	
L. hengduanshanensis-1	64.63	54.14	66.04	conserved	conserved	conserved	XXXXX	
L. hengduanshanensis-2	64.63	54.78	66.20	conserved	conserved	conserved	XXXXX	
L. hengduanshanensis-3	65.04	55.41	65.73	conserved	conserved	conserved	XXXXX	
L. hengduanshanensis-4	64.63	55.41	65.73	conserved	conserved	conserved	XXXXX	
L. hengduanshanensis-5	64.63	54.78	66.20	conserved	conserved	conserved	XXXXX	
L. hengduanshanensis-6	65.04	54.78	66.20	conserved	conserved	conserved	XXXXX	
L. hengduanshanensis-7	64.63	54.78	66.20	conserved	conserved	conserved	XXXXX	
L. hengduanshanensis-8	63.01	54.78	66.20	conserved	conserved	conserved	XXXXX	
L. hengduanshanensis-9	64.63	54.78	66.20	conserved	conserved	conserved	XXXXX	
L. hengduanshanensis-10	63.82	54.78	65.73	conserved	conserved	conserved	XXXXX	
L. hirta-1	61.22	54.78	64.35	conserved	conserved	conserved	XXXXX	
L. hirta-2	61.22	54.78	64.35	conserved	conserved	conserved	XXXXX	
L. hirta-3	61.22	54.78	64.35	conserved	conserved	conserved	XXXXX	
L. hirta-4	61.22	54.78	64.35	conserved	conserved	conserved	XXXXX	
L. hirta-5	61.22	54.78	64.35	conserved	conserved	conserved	XXXXX	
L. hirta-6	61.22	54.78	63.89	conserved	conserved	conserved	XXXXX	
L. hirta-7	61.22	54.78	63.89	conserved	conserved	conserved	XXXXX	
L. hirta-8	61.22	54.78	64.35	conserved	conserved	conserved	XXXXX	

L. hirta-9	61.22	54.78	64.35	conserved	conserved	conserved	XXXXX	
L. hispida-1	65.86	55.41	69.48	conserved	conserved	conserved	XXXXX	
L. hispida-2	65.86	55.41	69.48	conserved	conserved	conserved	XXXXX	
L. hispida-3	65.86	55.41	69.48	conserved	conserved	conserved	XXXXX	
L. hispida-4	65.86	55.41	69.01	conserved	conserved	conserved	XXXXX	
L. hispida-5	65.86	55.41	69.48	conserved	conserved	conserved	XXXXX	
L. hispida-6	65.86	56.05	69.48	conserved	conserved	conserved	XXXXX	
L. hispida-7	65.86	55.41	69.48	conserved	conserved	conserved	XXXXX	
L. hispida-8	66.27	55.41	69.48	conserved	conserved	conserved	XXXXX	
L. homoloba-1	65.20	54.78	67.61	conserved	conserved	conserved	XXXXX	
L. homoloba-2	65.20	54.78	67.61	conserved	conserved	conserved	XXXXX	
L. homoloba-3	65.60	54.78	67.61	conserved	conserved	conserved	XXXXX	
L. homoloba-4	65.60	54.78	67.61	conserved	conserved	conserved	XXXXX	
L. homoloba-5	65.20	54.78	67.61	conserved	conserved	conserved	XXXXX	
L. homoloba-6	65.20	54.78	68.08	conserved	conserved	conserved	XXXXX	
L. homoloba-7	65.20	54.78	67.61	conserved	conserved	conserved	XXXXX	
L. homoloba-8	65.20	54.78	67.61	conserved	conserved	conserved	XXXXX	
L. homoloba-9	65.20	54.78	67.61	conserved	conserved	conserved	XXXXX	
L. inschnica-2-1	63.71	54.78	66.67	conserved	conserved	conserved	XXXXX	
***L. inschnica*-2-2**	**61.48**	**52.23**	**63.85**	conserved	**nt-7**	**nt-8**	**XXX**-**X**	**pseudogene**
L. inschnica-2-3	63.71	54.78	66.67	conserved	conserved	conserved	XXXXX	
L. inschnica-2-4	63.71	54.78	66.67	conserved	conserved	conserved	XXXXX	
L. inschnica-2-5	63.71	54.78	66.67	conserved	conserved	conserved	XXXXX	
L. inschnica-2-6	63.71	55.41	66.67	conserved	conserved	conserved	XXXXX	
L. inschnica-2-7	63.71	54.78	66.67	conserved	conserved	conserved	XXXXX	
L. inschnica-2-8	63.71	54.78	66.67	conserved	conserved	conserved	XXXXX	
L. inschnica-2-9	63.71	54.78	67.14	conserved	conserved	conserved	XXXXX	
L. inschnica-2-10	64.11	54.78	66.67	conserved	conserved	conserved	XXXXX	
L. intermedia-1	63.37	54.78	63.38	conserved	conserved	conserved	XXXXX	
L. intermedia-2	62.96	53.50	63.85	conserved	conserved	conserved	XXXXX	
L. intermedia-3	62.96	54.78	63.38	conserved	conserved	conserved	XXXXX	
L. intermedia-4	62.96	54.78	63.38	conserved	conserved	conserved	XXXXX	
L. intermedia-5	62.55	54.78	63.38	conserved	conserved	conserved	XXXXX	
L. intermedia-6	63.37	54.78	63.38	conserved	conserved	conserved	XXXXX	
L. intermedia-7	62.96	54.78	63.38	conserved	conserved	conserved	XXXXX	
L. intermedia-8	62.96	54.78	63.38	conserved	conserved	conserved	XXXXX	
L. intermedia-9	62.96	53.50	63.85	conserved	conserved	conserved	XXXXX	
L. intermedia-10	62.96	54.78	63.38	conserved	conserved	conserved	XXXXX	
L. intermedia-11	62.96	54.78	63.38	conserved	conserved	conserved	XXXXX	
***L. jiangxiensis*-1-1**	**58.61**	**50.96**	**59.62**	**nt-16**	conserved	**conserved**	**XXXX**-	**pseudogene**
L. jiangxiensis-1-2	64.78	54.78	67.61	conserved	conserved	conserved	XXXXX	
L. jiangxiensis-1-3	64.78	54.78	65.26	conserved	conserved	conserved	XXXXX	
L. jiangxiensis-1-4	64.78	54.78	66.67	conserved	conserved	conserved	XXXXX	
L. jiangxiensis-1-5	64.78	54.78	66.67	conserved	conserved	conserved	XXXXX	
***L. jiangxiensis*-1-6**	**58.61**	54.03	66.67	conserved	**nt-1**	**all missing**	**XXX**--	**pseudogene**
L. jiangxiensis-1-7	65.18	54.78	68.08	conserved	conserved	conserved	XXXXX	
L. jiangxiensis-1-8	64.78	54.78	66.67	conserved	conserved	conserved	XXXXX	
L. jiangxiensis-1-9	64.78	54.78	66.67	conserved	conserved	conserved	XXXXX	
L. jiangxiensis-1-10	65.18	54.78	68.08	conserved	conserved	conserved	XXXXX	
L. jiangxiensis-2-1	64.37	54.78	66.67	conserved	conserved	conserved	XXXXX	
L. jiangxiensis-2-2	64.78	54.78	66.67	conserved	conserved	conserved	XXXXX	

L. jiangxiensis-2-3	64.78	54.78	66.67	conserved	conserved	conserved	XXXXX	
L. jiangxiensis-2-4	64.78	54.78	67.14	conserved	conserved	conserved	XXXXX	
***L. jiangxiensis*-2-5**	64.78	54.78	**63.38**	conserved	conserved	conserved	XXXXX	**pseudogene**
L. jiangxiensis-2-6	65.18	54.78	68.54	conserved	conserved	conserved	XXXXX	
L. jiangxiensis-2-7	65.18	54.78	68.54	conserved	conserved	conserved	XXXXX	
L. jiangxiensis-2-8	64.98	54.78	67.96	conserved	conserved	conserved	XXXXX	
L. jiangxiensis-2-9	65.59	54.78	68.54	conserved	conserved	conserved	XXXXX	
L. jiangxiensis-2-10	65.18	54.78	66.20	conserved	conserved	conserved	XXXXX	
L. juncea-3-1	64.52	54.78	66.67	conserved	conserved	conserved	XXXXX	
L. juncea-3-2	63.71	54.78	66.67	conserved	conserved	conserved	XXXXX	
L. juncea-3-3	63.71	54.78	66.67	conserved	conserved	conserved	XXXXX	
L. juncea-3-4	63.71	54.78	66.67	conserved	conserved	conserved	XXXXX	
L. juncea-3-5	63.31	54.78	66.20	conserved	conserved	conserved	XXXXX	
L. juncea-3-6	63.71	54.78	66.67	conserved	conserved	conserved	XXXXX	
L. lichiyuniae-3-1	65.18	54.78	68.08	conserved	conserved	conserved	XXXXX	
L. lichiyuniae-3-2	65.18	54.78	68.08	conserved	conserved	conserved	XXXXX	
L. lichiyuniae-3-3	65.18	54.78	67.61	conserved	conserved	conserved	XXXXX	
L. lichiyuniae-3-4	64.78	54.78	68.08	conserved	conserved	conserved	XXXXX	
***L. lichiyuniae*-3-5**	**60.08**	**51.59**	**58.69**	**nt-16**	**nt-7**	**conserved**	XXXXX	**pseudogene**
L. lichiyuniae-3-6	64.78	54.78	68.08	conserved	conserved	conserved	XXXXX	
L. lichiyuniae-3-7	65.18	54.78	68.08	conserved	conserved	conserved	XXXXX	
***L. lichiyuniae*-3-8**	65.18	54.78	**60.09**	conserved	conserved	conserved	**XXXX-**	**pseudogene**
L. lichiyuniae-3-9	65.18	54.78	68.08	conserved	conserved	conserved	XXXXX	
L. lichiyuniae-3-10	64.78	54.78	68.08	conserved	conserved	conserved	XXXXX	
L. maximowiczii-1-1	65.32	54.78	68.08	conserved	conserved	conserved	XXXXX	
L. maximowiczii-1-2	66.27	54.78	68.08	conserved	conserved	conserved	XXXXX	
L. maximowiczii-1-3	66.27	54.78	67.61	conserved	conserved	conserved	XXXXX	
L. maximowiczii-1-4	66.67	54.78	68.08	conserved	conserved	conserved	XXXXX	
L. maximowiczii-1-5	66.27	54.78	68.08	conserved	conserved	conserved	XXXXX	
L. maximowiczii-1-6	65.73	54.78	68.08	conserved	conserved	conserved	XXXXX	
L. maximowiczii-1-7	66.27	54.78	68.08	conserved	conserved	conserved	XXXXX	
L. maximowiczii-1-8	66.27	54.78	68.08	conserved	conserved	conserved	XXXXX	
L. maximowiczii-2-1	65.87	54.78	68.08	conserved	conserved	conserved	XXXXX	
L. maximowiczii-2-2	66.27	54.78	68.08	conserved	conserved	conserved	XXXXX	
L. maximowiczii-2-3	64.92	54.78	68.08	conserved	conserved	conserved	XXXXX	
L. maximowiczii-2-4	66.27	54.78	68.08	conserved	conserved	conserved	XXXXX	
L. maximowiczii-2-5	66.00	54.78	68.54	conserved	conserved	conserved	XXXXX	
L. maximowiczii-2-6	65.87	54.78	68.54	conserved	conserved	conserved	XXXXX	
L. maximowiczii-2-7	65.87	54.78	68.08	conserved	conserved	conserved	XXXXX	
L. maximowiczii-2-8	66.27	54.78	68.08	conserved	conserved	conserved	XXXXX	
L. maximowiczii-2-9	64.92	54.78	68.08	conserved	conserved	conserved	XXXXX	
L. maximowiczii-2-10	66.67	54.78	68.08	conserved	conserved	conserved	XXXXX	
L. melanantha-1	66.40	54.78	68.08	conserved	conserved	conserved	XXXXX	
L. melanantha-2	66.40	55.41	68.08	conserved	conserved	conserved	XXXXX	
L. melanantha-3	66.40	54.78	68.08	conserved	conserved	conserved	XXXXX	
L. melanantha-4	66.40	54.78	68.08	conserved	conserved	conserved	XXXXX	
L. melanantha-5	66.40	54.78	68.08	conserved	conserved	conserved	XXXXX	
L. melanantha-6	66.40	54.78	68.08	conserved	conserved	conserved	XXXXX	
L. melanantha-7	66.40	54.78	68.08	conserved	conserved	conserved	XXXXX	
L. patens-1	65.06	54.78	68.08	conserved	conserved	conserved	XXXXX	

***L. patens*-2**	**61.63**	54.14	**64.32**	conserved	conserved	conserved	XXXXX	**pseudogene**
L. patens-3	65.85	55.41	67.14	conserved	conserved	conserved	XXXXX	
***L. patens*-4**	**57.74**	54.14	**61.03**	conserved	conserved	conserved	**XXXX-**	**pseudogene**
***L. patens*-5**	**56.49**	**59.18**	**60.09**	**missing 4-16**	**all missing**	**nt-2**	**---XX**	**pseudogene**
***L. patens*-6**	**58.09**	**49.68**	**57.28**	**nt-16**	conserved	**nt-7**	**XX-XX**	**pseudogene**
L. patens-7	65.32	54.78	68.08	conserved	conserved	conserved	XXXXX	
L. patens-8	65.18	55.41	68.08	conserved	conserved	conserved	XXXXX	
L. patens-9	64.92	54.78	67.61	conserved	conserved	conserved	XXXXX	
***L. patens*-10**	**54.20**	**42.31**	**47.02**	**nt-9,12,16**	**nt-6,14**	**nt-8,9,10**	**-----**	**pseudogene**
L. pilosa-1	64.78	54.78	67.14	conserved	conserved	conserved	XXXXX	
L. pilosa-2	64.78	54.78	66.67	conserved	conserved	conserved	XXXXX	
L. pilosa-3	64.78	54.78	67.14	conserved	conserved	conserved	XXXXX	
L. pilosa-4	64.78	54.78	66.67	conserved	conserved	conserved	XXXXX	
L. pilosa-5	64.78	54.78	66.67	conserved	conserved	conserved	XXXXX	
L. pilosa-6	64.78	54.78	66.67	conserved	conserved	conserved	XXXXX	
L. pilosa-7	65.18	54.78	66.67	conserved	conserved	conserved	XXXXX	
L. pilosa-8	65.18	54.78	66.67	conserved	conserved	conserved	XXXXX	
L. pilosa-9	64.78	54.78	66.67	conserved	conserved	conserved	XXXXX	
L. potaninii-3-1	63.71	54.78	67.61	conserved	conserved	conserved	XXXXX	
L. potaninii-3-2	63.31	54.14	67.61	conserved	conserved	conserved	XXXXX	
L. potaninii-3-3	63.71	54.78	67.61	conserved	conserved	conserved	XXXXX	
L. potaninii-3-4	62.90	54.78	67.14	conserved	conserved	conserved	XXXXX	
L. potaninii-3-5	63.05	54.78	67.61	conserved	conserved	conserved	XXXXX	
L. potaninii-3-6	63.71	54.78	67.61	conserved	conserved	conserved	XXXXX	
L. potaninii-3-7	63.31	54.78	67.14	conserved	conserved	conserved	XXXXX	
L. potaninii-3-8	63.31	54.78	67.14	conserved	conserved	conserved	XXXXX	
L. repens-1	63.37	54.49	64.79	conserved	conserved	conserved	XXXXX	
L. repens-2	62.96	54.78	64.32	conserved	conserved	conserved	XXXXX	
L. repens-3	62.96	54.78	64.79	conserved	conserved	conserved	XXXXX	
L. repens-4	62.96	54.78	64.79	conserved	conserved	conserved	XXXXX	
L. repens-5	63.37	54.49	64.79	conserved	conserved	conserved	XXXXX	
L. repens-6	62.96	54.78	64.32	conserved	conserved	conserved	XXXXX	
L. repens-7	62.96	54.78	64.32	conserved	conserved	conserved	XXXXX	
L. repens-8	62.96	54.78	64.32	conserved	conserved	conserved	XXXXX	
L. repens-9	62.96	54.78	64.32	conserved	conserved	conserved	XXXXX	
L. stuevei-1	62.96	54.78	63.38	conserved	conserved	conserved	XXXXX	
L. stuevei-2	62.96	54.78	63.38	conserved	conserved	conserved	XXXXX	
L. stuevei-3	62.20	54.78	63.38	conserved	conserved	conserved	XXXXX	
L. stuevei-4	62.96	54.78	63.38	conserved	conserved	conserved	XXXXX	
L. stuevei-5	62.96	54.78	63.38	conserved	conserved	conserved	XXXXX	
L. stuevei-6	62.55	54.78	63.38	conserved	conserved	conserved	XXXXX	
L. stuevei-7	62.96	54.78	63.38	conserved	conserved	conserved	XXXXX	
L. stuevei-8	62.96	54.78	63.38	conserved	conserved	conserved	XXXXX	
L. stuevei-9	62.55	54.78	63.38	conserved	conserved	conserved	XXXXX	
L. stuevei-10	62.96	54.78	63.38	conserved	conserved	conserved	XXXXX	
L. tomentosa-1-1	62.03	55.41	63.30	conserved	conserved	conserved	XXXXX	
L. tomentosa-1-2	62.03	55.41	63.30	conserved	conserved	conserved	XXXXX	
L. tomentosa-1-3	62.45	55.41	63.30	conserved	conserved	conserved	XXXXX	
L. tomentosa-1-4	62.03	55.41	63.30	conserved	conserved	conserved	XXXXX	
L. tomentosa-1-5	62.03	55.41	63.30	conserved	conserved	conserved	XXXXX	

L. tomentosa-1-6	62.03	55.41	63.30	conserved	conserved	conserved	XXXXX	
L. tomentosa-1-7	61.60	55.41	63.30	conserved	conserved	conserved	XXXXX	
L. tomentosa-1-8	62.03	55.41	63.30	conserved	conserved	conserved	XXXXX	
L. tomentosa-1-9	62.03	55.41	63.30	conserved	conserved	conserved	XXXXX	
L. tomentosa-1-10	62.03	55.41	63.30	conserved	conserved	conserved	XXXXX	
L. violacea-1	62.96	54.78	64.32	conserved	conserved	conserved	XXXXX	
L. violacea-2	62.96	54.78	64.32	conserved	conserved	conserved	XXXXX	
L. violacea-3	62.96	54.78	64.32	conserved	conserved	conserved	XXXXX	
L. violacea-4	62.96	54.78	64.32	conserved	conserved	conserved	XXXXX	
L. violacea-5	62.96	54.14	64.32	conserved	conserved	conserved	XXXXX	
L. violacea-6	62.96	54.78	64.32	conserved	conserved	conserved	XXXXX	
L. violacea-7	62.96	54.78	64.32	conserved	conserved	conserved	XXXXX	
L. violacea-8	62.96	54.78	64.32	conserved	conserved	conserved	XXXXX	
L. violacea-9	62.96	54.78	64.32	conserved	conserved	conserved	XXXXX	
L. violacea-10	62.96	54.78	64.32	conserved	conserved	conserved	XXXXX	
L. virgata-2-1	60.34	54.78	62.91	conserved	conserved	conserved	XXXXX	
L. virgata-2-2	60.76	54.78	62.91	conserved	conserved	conserved	XXXXX	
L. virgata-2-3	60.76	54.78	62.91	conserved	conserved	conserved	XXXXX	
L. virgata-2-4	60.76	54.78	62.91	conserved	conserved	conserved	XXXXX	
L. virgata-2-5	60.76	54.78	62.91	conserved	conserved	conserved	XXXXX	
L. virgata-2-6	60.76	54.78	62.91	conserved	conserved	conserved	XXXXX	
L. virgata-2-7	60.76	54.78	62.91	conserved	conserved	conserved	XXXXX	
L. virgata-2-8	60.76	54.78	62.91	conserved	conserved	conserved	XXXXX	
L. virgata-2-9	61.18	54.78	63.38	conserved	conserved	conserved	XXXXX	
L. virgata-2-10	60.76	54.78	62.91	conserved	conserved	conserved	XXXXX	
L. virginica-1	62.96	54.78	63.85	conserved	conserved	conserved	XXXXX	
L. virginica-2	62.96	54.78	63.85	conserved	conserved	conserved	XXXXX	
L. virginica-3	62.96	54.78	63.85	conserved	conserved	conserved	XXXXX	
L. virginica-4	62.96	54.78	63.85	conserved	conserved	conserved	XXXXX	
L. virginica-5	62.96	54.78	63.85	conserved	conserved	conserved	XXXXX	
L. virginica-6	62.96	54.78	63.85	conserved	conserved	conserved	XXXXX	
L. virginica-7	62.96	54.78	63.85	conserved	conserved	conserved	XXXXX	
L. virginica-8	62.96	54.78	63.85	conserved	conserved	conserved	XXXXX	
L. virginica-9	62.96	54.78	63.85	conserved	conserved	conserved	XXXXX	
L. wilfordii-1-1	65.32	54.78	68.54	conserved	conserved	conserved	XXXXX	
L. wilfordii-1-2	65.32	54.78	68.54	conserved	conserved	conserved	XXXXX	
L. wilfordii-1-3	64.92	54.78	68.54	conserved	conserved	conserved	XXXXX	
L. wilfordii-1-4	65.32	55.41	68.08	conserved	conserved	conserved	XXXXX	
L. wilfordii-1-5	65.73	54.78	68.08	conserved	conserved	conserved	XXXXX	
L. wilfordii-1-6	65.73	55.41	67.61	conserved	conserved	conserved	XXXXX	
***L. wilfordii*-1-7**	65.73	53.50	**60.56**	conserved	conserved	conserved	**XXXX-**	**pseudogene**
L. wilfordii-1-8	65.99	54.78	68.08	conserved	conserved	conserved	XXXXX	
L. wilfordii-1-9	65.32	54.78	68.08	conserved	conserved	conserved	XXXXX	
L. wilfordii-1-10	65.32	54.78	68.08	conserved	conserved	conserved	XXXXX	
***L. wilfordii*-2-1**	**61.89**	**51.92**	**60.09**	**nt-14**	conserved	**nt-4**	**XXXX-**	**pseudogene**
L. wilfordii-2-2	65.99	54.78	68.54	conserved	conserved	conserved	XXXXX	
***L. wilfordii*-2-3**	63.93	54.78	**62.91**	conserved	conserved	conserved	XXXXX	**pseudogene**
L. wilfordii-2-4	65.99	54.78	68.54	conserved	conserved	conserved	XXXXX	
L. wilfordii-2-5	65.73	54.78	68.08	conserved	conserved	conserved	XXXXX	
L. wilfordii-2-6	65.99	54.78	68.54	conserved	conserved	conserved	XXXXX	
***L. wilfordii*-2-7**	**59.43**	**51.92**	**59.62**	**nt-14**	conserved	**nt-4**	**XXXX-**	**pseudogene**

***L. wilfordii*-2-8**	**46.98**	**42.31**	**47.62**	**nt-8,9,12,16**	**nt-6,14**	**nt-8,9,10**	-----	**pseudogene**
***L. wilfordii*-2-9**	**59.84**	**51.92**	**60.09**	**nt-14**	conserved	**nt-4**	**XXXX-**	**pseudogene**
L. wilfordii-3-1	66.27	54.78	68.08	conserved	conserved	conserved	XXXXX	
L. wilfordii-3-2	65.87	54.78	68.08	conserved	conserved	conserved	XXXXX	
***L. wilfordii*-3-3**	**54.51**	**51.59**	**59.62**	**nt-2,11**	**nt-9**	conserved	**X-XXX**	**pseudogene**
L. wilfordii-3-4	65.87	54.78	68.08	conserved	conserved	conserved	XXXXX	
L. wilfordii-3-5	65.87	54.78	68.08	conserved	conserved	conserved	XXXXX	
L. wilfordii-3-6	65.87	54.78	68.08	conserved	conserved	conserved	XXXXX	
L. wilfordii-3-7	66.27	54.14	68.08	conserved	conserved	conserved	XXXXX	
